# Synthesis of benzothiazole-appended bis-triazole-based structural isomers with promising antifungal activity against *Rhizoctonia solani*†

**DOI:** 10.1039/d2ra04465j

**Published:** 2022-08-30

**Authors:** Ravindra Kumar Upadhyay, Keshav Kumar Saini, Nidhi Deswal, Tejveer Singh, Kailash Pati Tripathi, Parshant Kaushik, Najam Akhtar Shakil, Alok Chandra Bharti, Rakesh Kumar

**Affiliations:** Department of Chemistry, University of Delhi Delhi-110007 India rakeshkp@email.com; Molecular Oncology Laboratory, Department of Zoology, University of Delhi, School of Life Sciences, JNU-Delhi India; Division of Agricultural Chemicals, Indian Agricultural Research Institute New Delhi India; Molecular Oncology Laboratory, Department of Zoology, University of Delhi Delhi-110007 India

## Abstract

In order to explore new antifungal agrochemicals, we reported the synthesis of two series 5a–f, 6 and 7a–f, 8 of benzothiazole-appended bis-triazole derivative-based structural isomers using a molecular hybridization approach. The synthesized compounds were tested for fungal growth inhibition against the plant pathogen *Rhizoctonia solani*. All the synthesized compounds showed excellent antifungal activity in their minimum concentrations (10–0.62 μM). Among all the synthetics, compounds 5b (ED_50_: 2.33 μM), 5f (ED_50_: 0.96 μM), and 7f (ED_50_: 1.48 μM) exerted a superior inhibitory effect in comparison to the commercially available fungicide, hexaconazole (ED_50_: 2.44 μM). The binding interactions of the active compounds 5f, 7f, 6, and 8 within the active site of the sterol 14α-demethylase enzyme were studied with the help of molecular docking studies. The studies revealed that these hybrid pharmacophores could be used as an important intermediate to demonstrate new structural isomer-based fungicides.

## Abstract 2

Two novel series (5a–f, 6 and 7a–f, 8) of benzothiazole-appended bis-triazole derivative-based fungicides were prepared and their antifungal activity evaluated against plant pathogen *Rhizoctonia solani*. It was found that compound 5f is the most potent fungicide.

## Graphical abstract

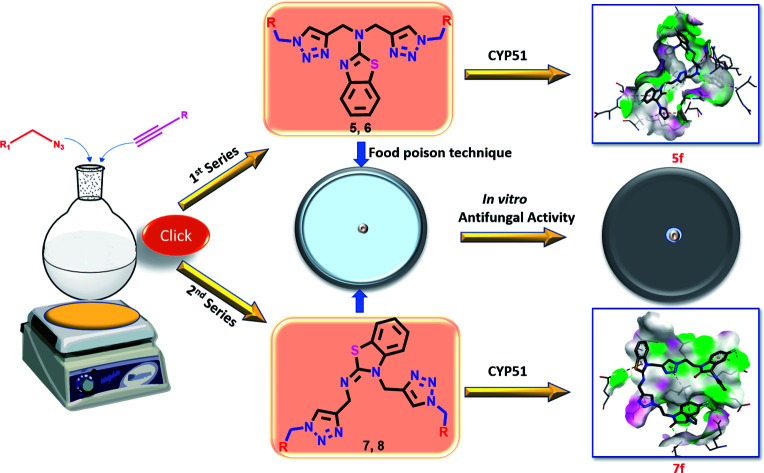

## 1. Introduction

The agricultural industry is scrambling at a great pace to meet the requirements of food for the exponentially growing world population. One of the major hurdles in fulfilling global food security is debilitating plant diseases.^1,2^ These diseases are responsible for both low yield and low quality of food grains, leading to a substantial economic loss for the country.^3^ Phytopathogenic fungi, a group of many plant pathogens, cause a wide variety of crop diseases. According to the statistics of the World Harvest of Food and Agriculture Organization of the UN (2009–2010), pathogenic fungi, particularly *Rhizoctonia solani* Kuhn fungus, globally affect five major crops, namely rice, wheat, maize, potatoes, and soybean.^4^ This fungus gives rise to sheath blight disease (oriental sheath) and leaf blight in rice that causes stunting, wilting of plant leaves, and possibly plant death.^5^ It is well documented that the permeability, fluidity, and viability of the fungal cells are maintained mainly by sterols. Predominantly, ergosterol (5,7-diene oxysterol) found in most of the fungal cell membranes has become an emerging target of the majority of clinically used therapeutics.^6^

To override the problem of crop loss caused due to fungal diseases, several fungicides are available in the global market that target the fungal cell membrane to disrupt its cell wall.^7^ Several other drugs under various stages of clinical trials also give an effective therapeutic choice to treat fungus-generated diseases. The increased chemical burden for controlling fungal diseases to improve the yield of crops has led to multiple drug resistance, crop toxicity, soil infertility, and environmental pollution.^8,9^ Therefore, the development of a new environment-friendly class of drugs possessing more bioactive sites is needed to be more effective against plant fungal infections with lower health risks.^10^ Herein, the concept of molecular hybridization plays a vital role in developing a coherent design of novel multitarget hybrid antifungal agents. Combined scaffolds probably propose some leads in minimizing drug resistance and enhancing their biological potency.

**Fig. 1:**
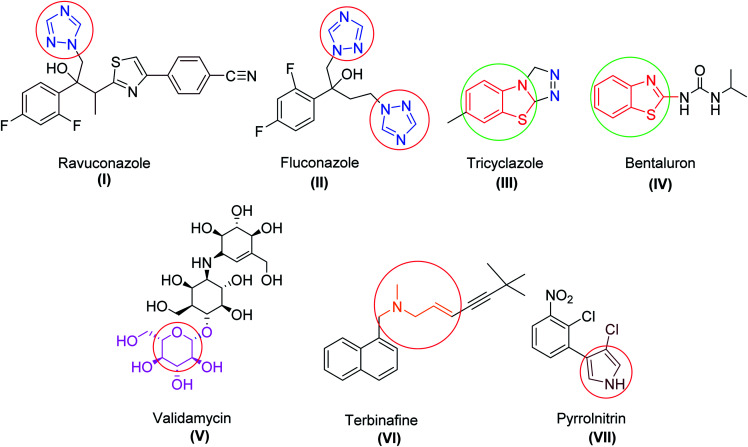
Structures of some antifungal drugs (I–VII).

Exploration of novel and potent antifungal agrochemicals can be achieved by the optimization and modification of the existing agents by minimizing their side effects. Many reports of available fungicides (Fig. 1) containing specific bioactive moieties, like alkyl amine, thiazole, benzothiazole, triazole, polyene, isatin, and sugar, have been found to inhibit the functioning of fungal cells.^11–15^ Apart from the antifungal activity, these heterocyclic scaffolds are also reported as privileged fragments due to their high affinity toward various biological targets in modern medicinal chemistry.^16–19^

**Fig. 2:**
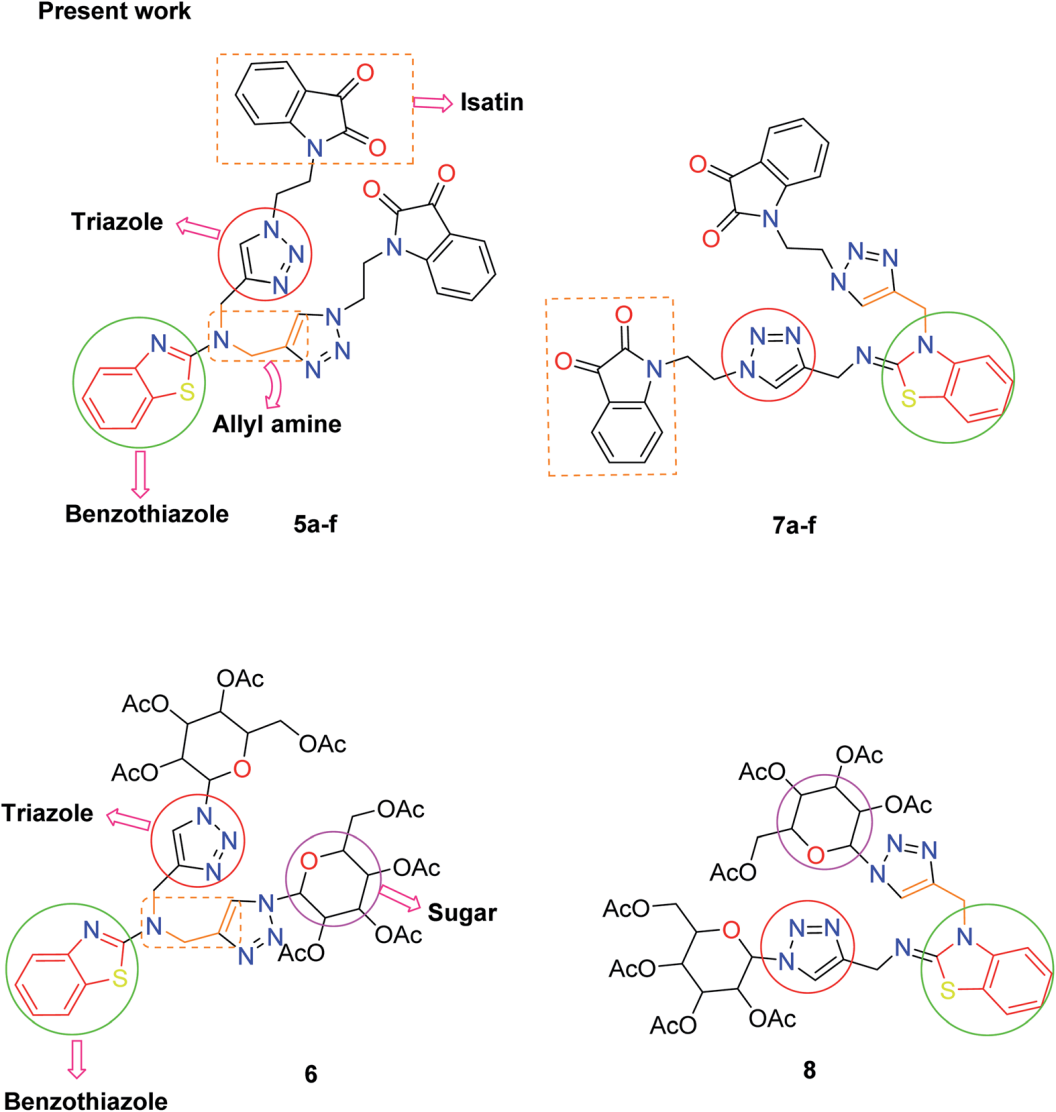
Design protocol of the target compounds.

There is considerable increasing interest in triazole moieties as these are known to block the active site of the 14α-demethylase enzyme, which is responsible for the biosynthesis of ergosterol.^20^ This inhibition of ergosterol biosynthesis leads to the suppression of fungal growth.^21^ Based on these findings, some novel hybrid pharmacophores containing triazole, pyrrole, benzothiazole, sugar, and isatin motifs have been formatted within a single molecule to obtain globally demanded antifungal agents (Fig. 2).

The relationship between chemical structure and biological activity is used to understand the effect of the insertion of new chemical entities in the basic pharmacophores to obtain more potent and more specific drugs. In this regard, structure and activity relationship (SAR) studies have been utilized to get a clear idea concerning the mechanism of action of the drugs.^22^ Herein, we report the synthesis of benzothiazole-appended bis-triazole derivatives based on structural isomers and the comparison of their potency against *Rhizoctonia solani* with the help of *in silico* as well as *in vitro* studies. It is anticipated that these synthesized candidates will lead to significant developments in the field of agrochemicals.

## 2. Results and discussion

### 2.1. Chemistry

**Scheme 1:**
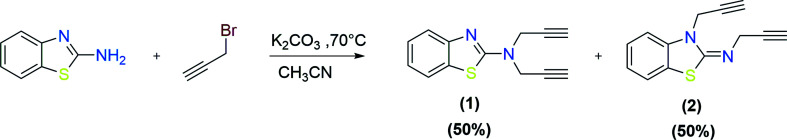
Synthesis of bis-propargylated 2-aminobenzothiazoles.

**Scheme 2:**
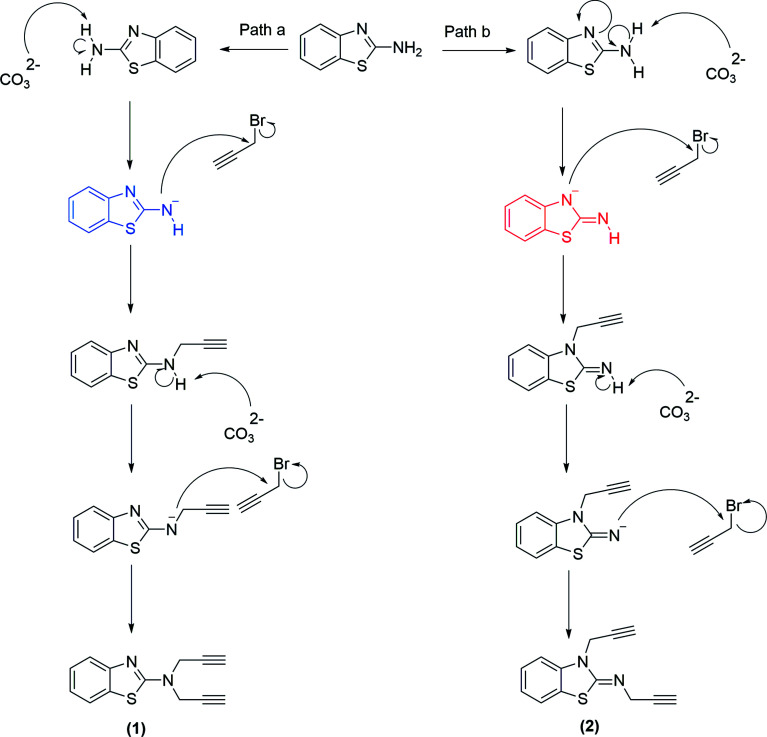
A possible mechanism for the synthesis of compounds 1 and 2.

The methodology for the formation of benzothiazole-appended bis-triazole derivatives involved two steps. In the first step, 2-aminobenzothiazole was allowed to react with propargyl bromide in the presence of K_2_CO_3_ in acetonitrile at 70 °C to afford two isomers, 1 and 2, in 1 : 1 yield, as shown in Scheme 1. The reaction was further screened for different solvent systems to optimize the reaction conditions as shown in Table 1. Among all the solvents, DMF was found to be an appropriate solvent in terms of yield for the present reaction (Scheme 1 and Table 1). The mechanistic pathway for the formation of isomers, 1 and 2 from 2-aminobenzothiazole is shown in Scheme 2.

**Table 1:** Optimization of the reaction conditions of Scheme 1 for the synthesis of compounds 1 and 2

Entry	Solvent	Base	Temp (°C)	Yield (%)
i	THF	K_2_CO_3_	70	No reaction
**ii**	**ACN**	**K**_**2**_ **CO**_**3**_	**70**	**50 : 50**
iii	DMF	K_2_CO_3_	70	95 : 5
iv	DMF	NaH	70	90 : 10
v	Ethanol : water	K_2_CO_3_	70	No reaction
vi	Dioxane	K_2_CO_3_	70	No reaction

**Table 2:** Structures of the synthesized benzothiazole-appended bis-triazole derivatives 5a–f, and 6

S. N.	Structure of compounds	Yields (%)	Melting point (°C)
1	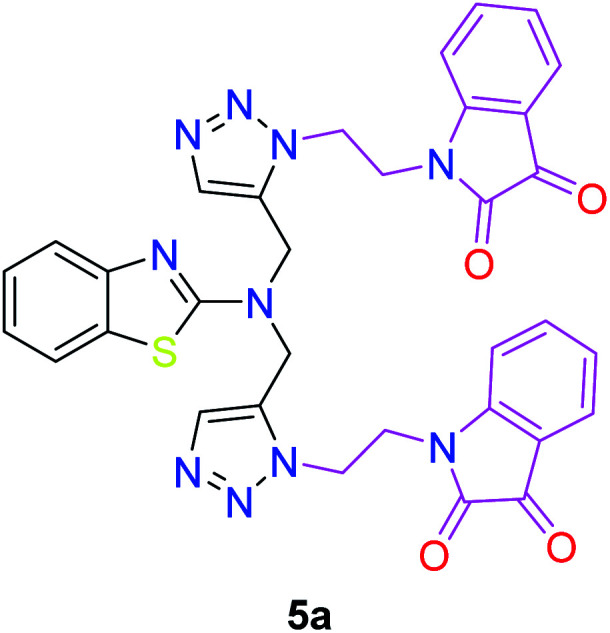	78	154–156
2	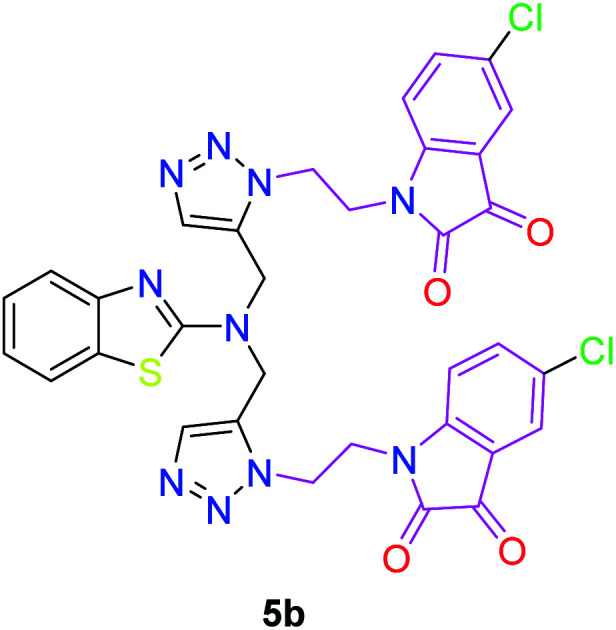	80	156–158
3	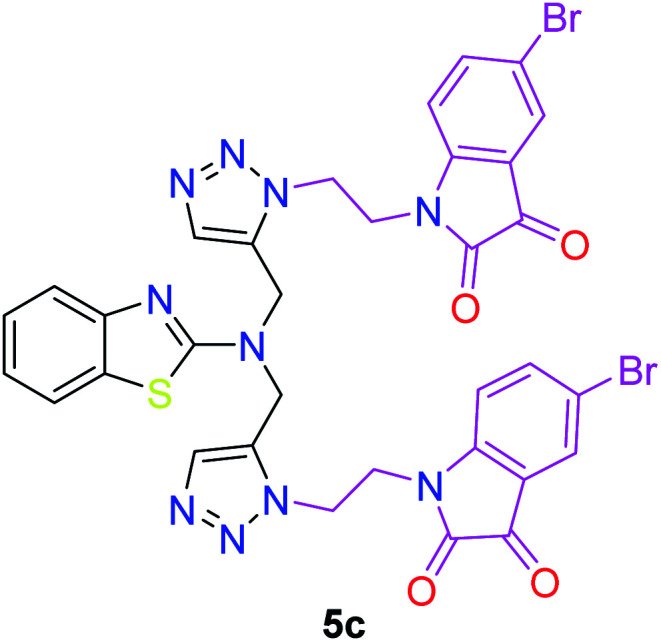	90	161–164
4	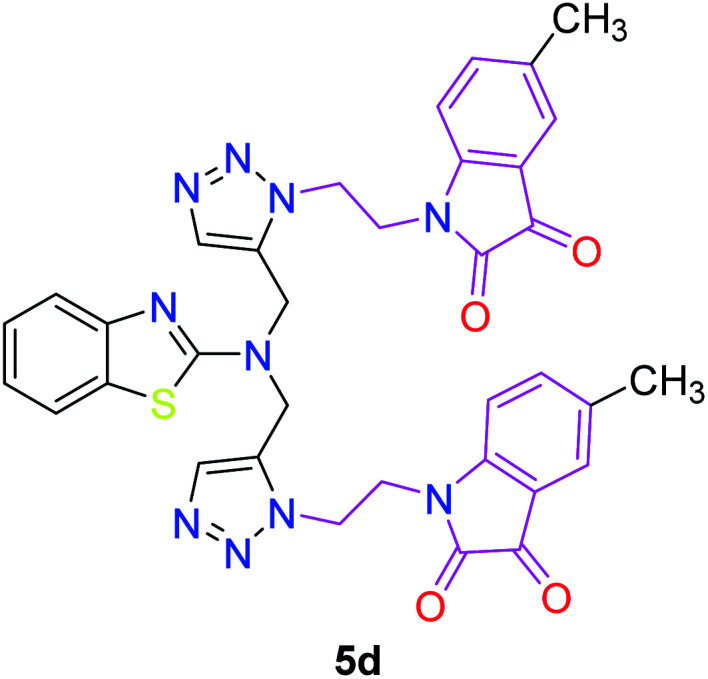	89	185–187
5	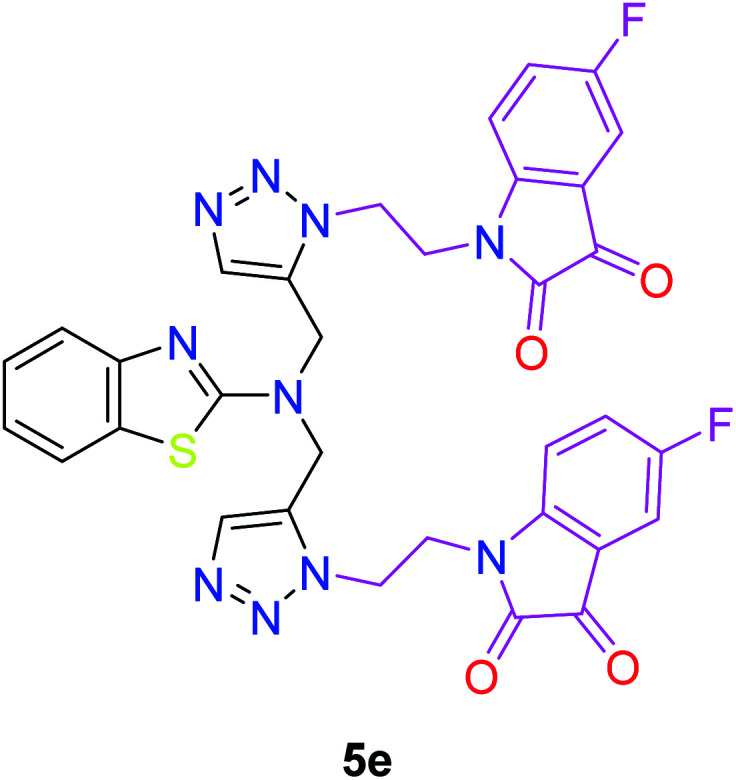	90	171–172
6	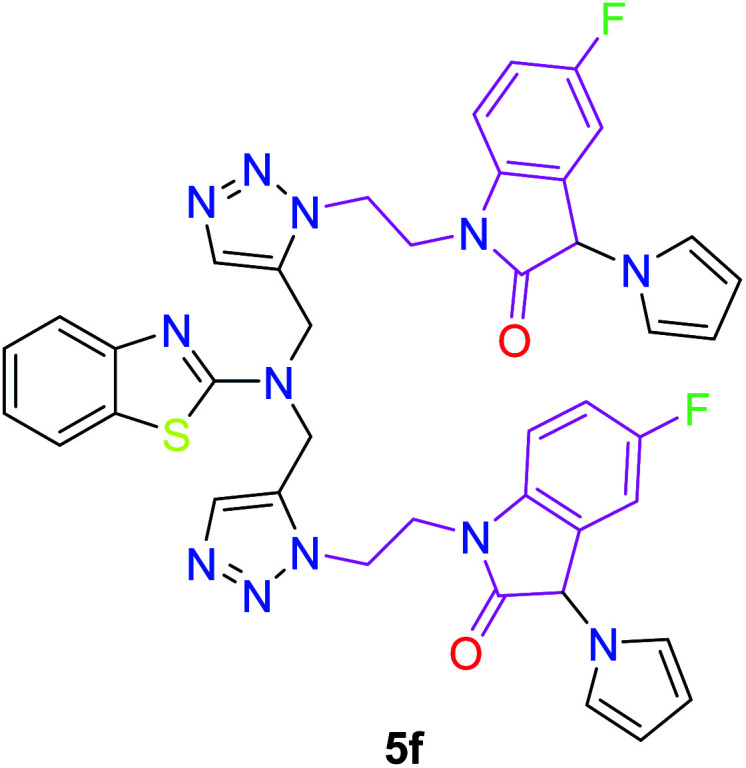	70	171–172
7	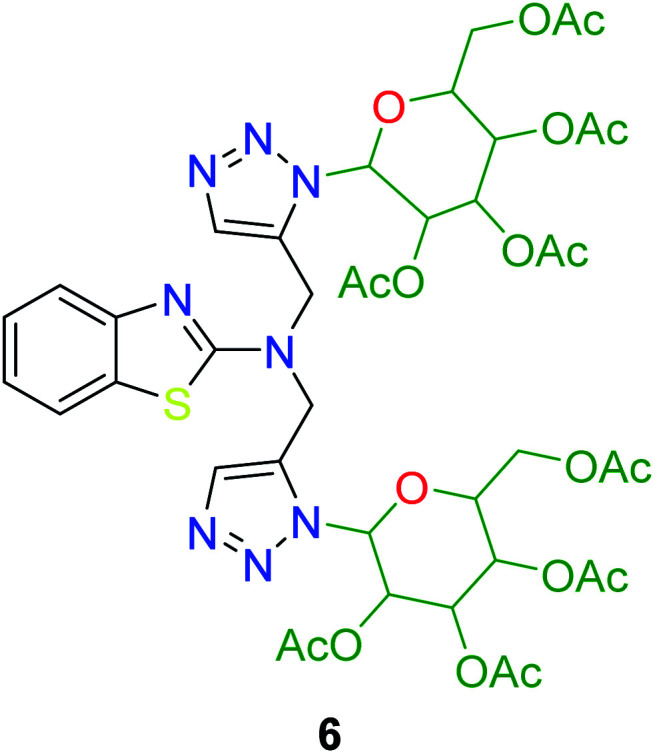	85	193–197

**Scheme 3:**
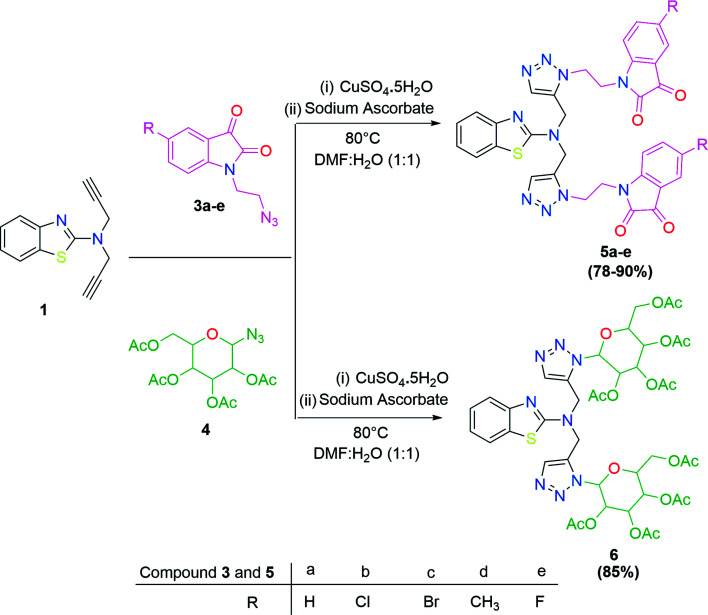
Synthesis of benzothiazole-appended bis-triazole derivatives 5a–e and 6.

**Scheme 4:**
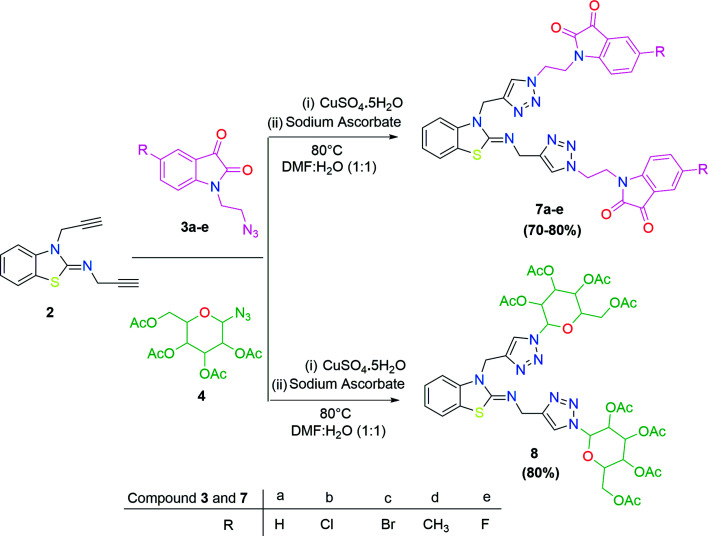
Synthesis of benzothiazole-appended bis-triazole derivatives 7a–e and 8.

**Scheme 5:**
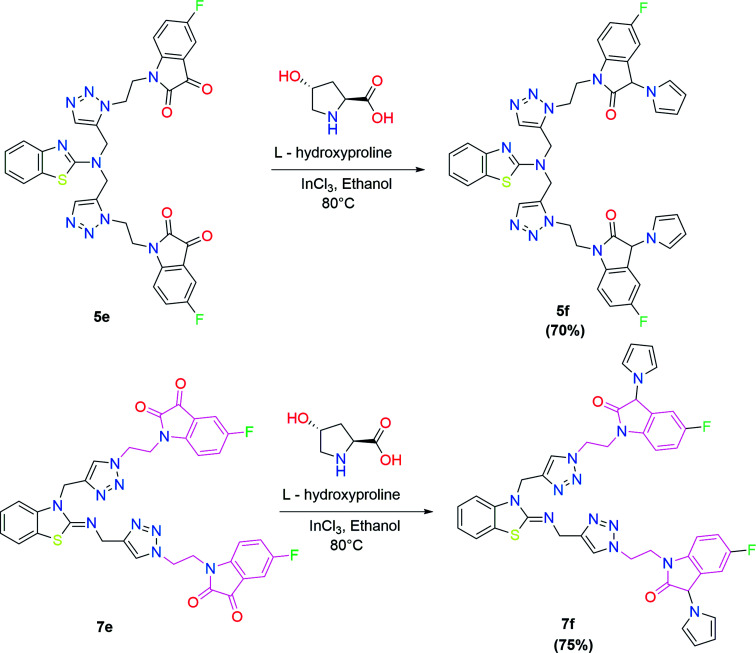
Synthesis of benzothiazole-appended bis-triazole derivatives containing 3-pyrrolylisatin 5f and 7f from 5e and 7e, respectively.

In the second step, the benzothiazole-appended bis-triazole derivatives 5a–e, 6 and 7a–e, 8 were synthesized *via* the click chemistry approach as depicted in Schemes 3 and 4, respectively. The azides of isatin (3a–e) and acyl-protected d-glucose (4) were prepared by the experimental conditions reported in the literature.^23^ These compounds were further treated with alkynes 1 and 2 in the presence of copper sulphate and sodium ascorbate in the different solvent systems at 80 °C to afford the corresponding desired derivatives. Furthermore, 5e and 7e were refluxed with l-hydroxyproline in ethanol in the presence of InCl_3_ for 3 h to get the corresponding derivatives 5f and 7f, as shown in Scheme 5.

The structures of all of the synthetics 5a–f, 6 and 7a–f, 8 (Tables 2 and 3) were confirmed by their spectral analysis. In the ^1^H NMR spectrum of the compound 5a, a singlet was observed at *δ* 4.3 ppm for 4 protons corresponding to the methylene group attached to the nitrogen(s) of the amino benzothiazole ring. Two triplets at *δ* 4.5 ppm and *δ* 4.6 ppm for 4 protons were assigned to the methylene group linked with the isatin ring. The characteristic peak appeared as a singlet at *δ* 8.33 ppm for two protons for the triazole hydrogen. The ^13^C NMR spectrum of compound 5a showed a peak at *δ* 180 ppm, which was assigned to the C=O (keto) carbon of the isatin ring.

**Table 3:** Structures of the synthesized b-enzothiazole-appended bis-triazole derivatives 7a–f, and 8

S. N.	Structure of compounds	Yields (%)	Melting point (°C)
1	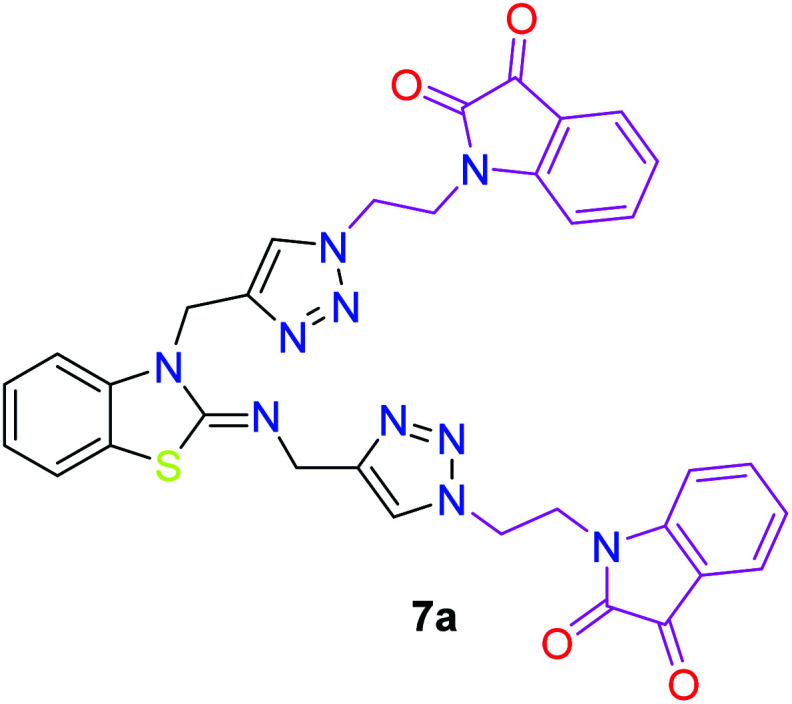	70	165–168
2	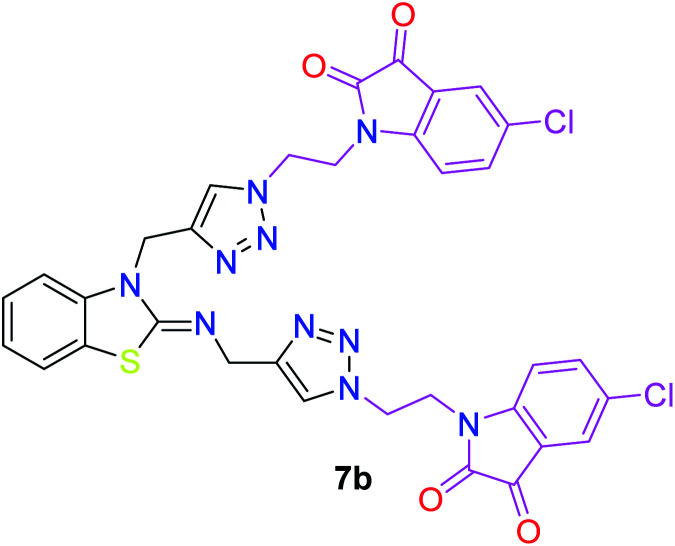	75	175–177
3	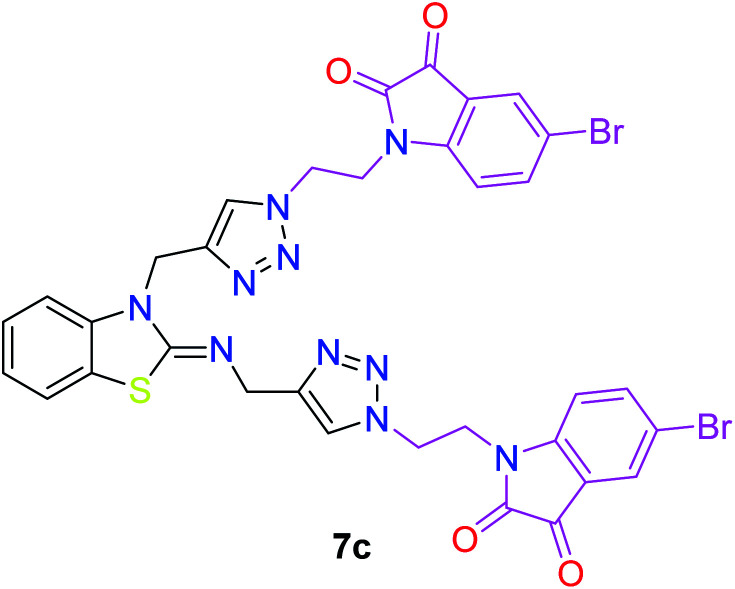	80	168–171
4	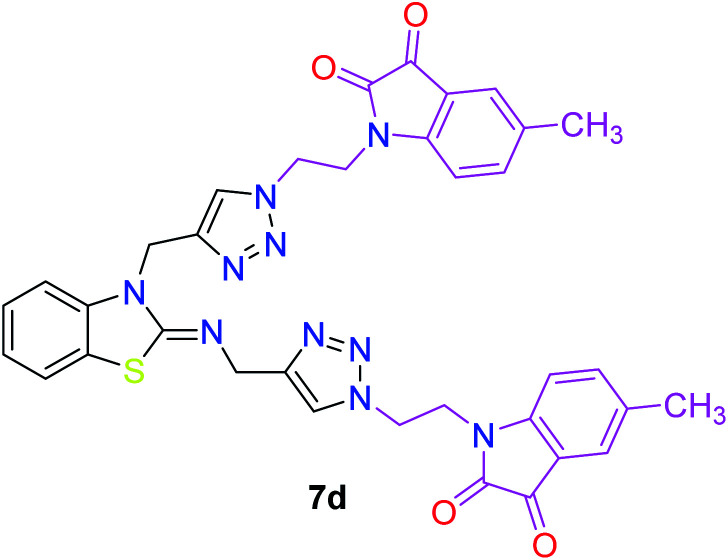	75	167–169
5	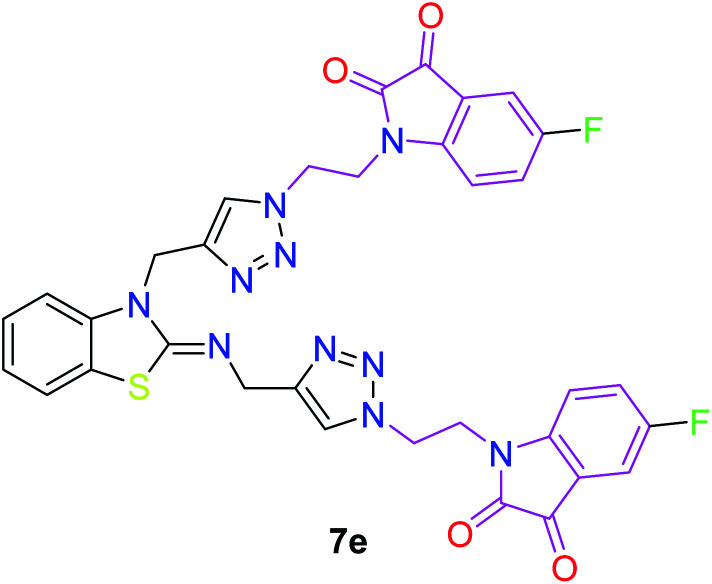	76	175–177
6	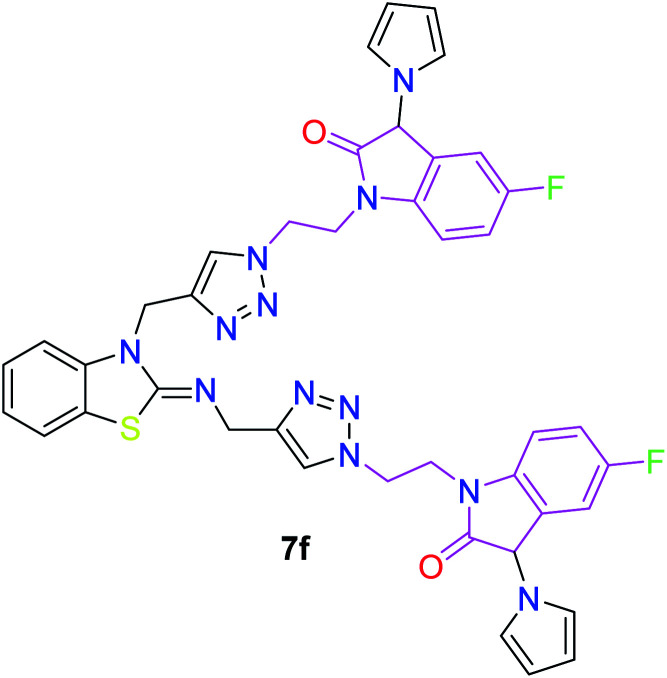	75	174–175
7	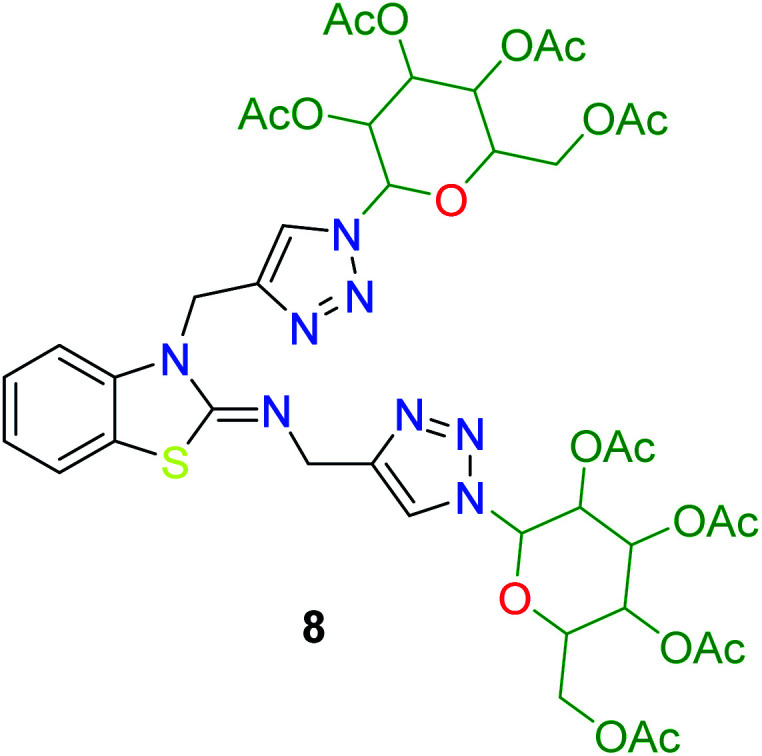	80	197–199

**Fig. 3:**
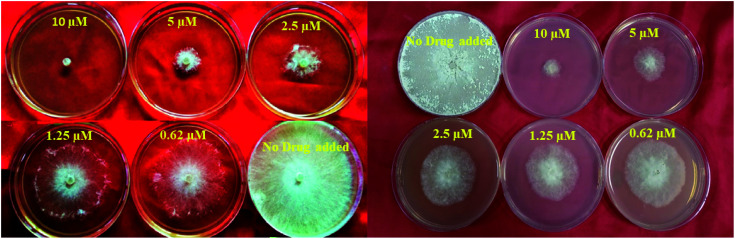
*In vitro* studies of the synthesized compounds at various concentrations using PDA culture media.

**Fig. 4:**
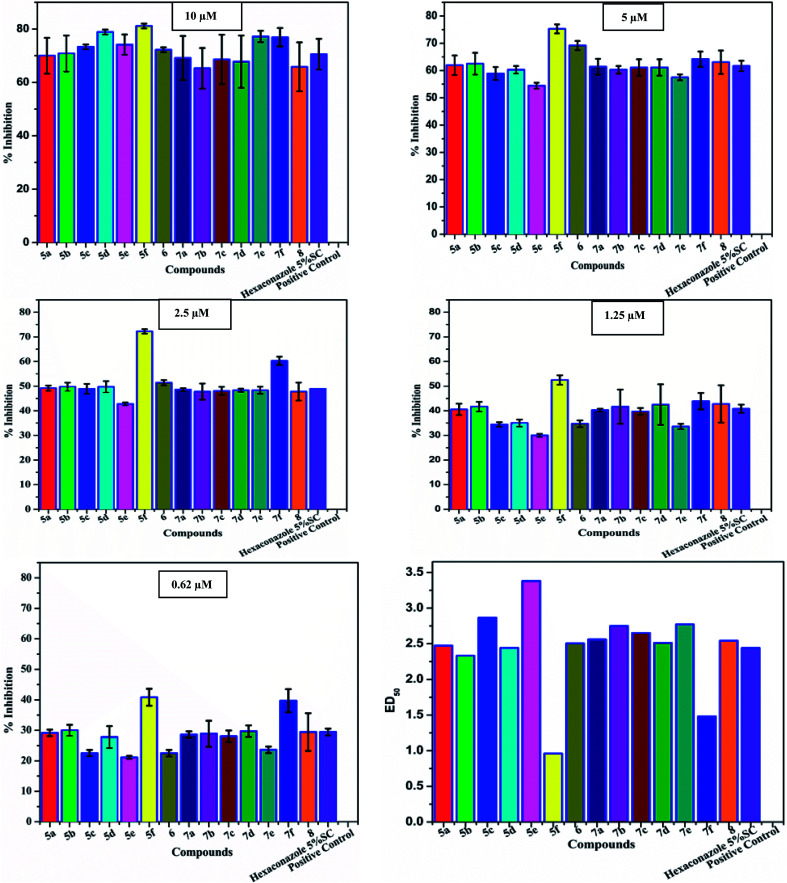
Antifungal evaluation against plant pathogen *Rhizoctonia solani* using 5a–f, 6 and 7a–f, 8 from 10 μM to 0.62 μM.

The peak appearing at *δ* 168 ppm was assigned to the imine carbon present in the benzothiazole ring. The peaks that appeared at *δ* 44.2 ppm and *δ* 47.3 ppm were assigned to methylene carbons. For compound 7a, the two singlets observed at *δ* 4.1 and *δ* 4.2 ppm for 4 protons corresponded to the methylene groups attached to both nitrogens present in the amino benzothiazole ring. Mass spectrometry data for both isomers 5a and 7a showed the calculated mass (M + H) ion of 659.1, satisfying the observed mass of 659.1. The structures of all of the synthesized compounds are shown in Fig. 3 and 4.

### 2.2. Pharmacology

#### 2.2.1 Antifungal potential and structure–activity relationship

The newly synthesized compounds 5a–f, 6 and 7a–f, 8 were screened for their antifungal activity against the plant pathogen *Rhizoctonia solani*. ED_50_ values were used as the effective concentration measurement required to inhibit 50% growth of the fungus and determined by at least four separate tests. The obtained results revealed that all compounds displayed excellent antifungal activity against *Rhizoctonia solani* at five concentrations (10 μM, 5 μM, 2.5 μM, 1.25 μM, and 0.62 μM) (Fig. 3 and Table 4).

**Table 4:** Percent inhibition of all the treated compounds at different concentrations

Compounds	10 μM	5 μM	2.5 μM	1.25 μM	0.62 μM
5a	70.00 ± 6.7	61.94 ± 3.6	49.16 ± 1.1	40.55 ± 2.3	29.16 ± 1.1
5b	**70.83** ± 6.8	**62.50** ± 4.0	**49.72** ± 1.7	**41.66** ± 1.9	**30.00** ± 1.8
5c	73.33 ± 0.9	58.88 ± 2.4	48.88 ± 2.0	34.44 ± 0.9	22.50 ± 1.1
5d	72.22 ± 0.9	69.16 ± 1.4	51.38 ± 2.3	34.72 ± 1.4	22.50 ± 3.6
5e	74.16 ± 2.5	54.44 ± 1.6	42.77 ± 1.4	30.00 ± 1.6	21.11 ± 1.6
5f	**81.11** ± 0.9	**75.27** ± 1.7	**72.22** ± 0.9	**52.50** ± 1.9	**40.83** ± 2.8
6	78.88 ± 0.9	60.27 ± 1.7	49.72 ± 1.1	35.00 ± 1.4	27.77 ± 1.1
7a	69.16 ± 8.2	61.38 ± 2.9	48.61 ± 0.6	40.27 ± 0.6	28.61 ± 1.1
7b	65.27 ± 7.6	60.27 ± 1.4	47.77 ± 3.3	41.66 ± 6.9	28.88 ± 4.3
7c	68.61 ± 9.3	61.11 ± 3.0	48.05 ± 1.7	39.72 ± 1.4	28.05 ± 1.9
7d	65.83 ± 9.8	63.05 ± 3.0	47.77 ± 0.6	42.77 ± 8.2	29.44 ± 1.9
7e	77.22 ± 2.1	57.5 ± 1.1	48.33 ± 1.4	33.61 ± 1.1	23.61 ± 1.1
7f	**76.94** ± 3.4	**64.16** ± 2.8	**60.27** ± 1.7	**43.88** ± 3.3	**39.72** ± 3.8
8	67.77 ± 9.2	61.11 ± 4.3	48.33 ± 3.6	42.50 ± 7.6	29.72 ± 6.2
**Hexaconazole 5% SC**	**70.55** ± 5.8	**61.66** ± 1.9	**48.88** ± 0.0	**40.83** ± 1.7	**29.44** ± 1.1

The structure–activity relationship (SAR) studies suggested that compounds 5f, 7b, and 7f exhibited excellent inhibitory effects on fungal growth compared to the commercially available fungicide hexaconazole (Table 5 and Fig. 4). However, compound 5f displayed the highest antifungal activity and was approximately three times more potent than hexaconazole in this study. In general, the results of antifungal activity showed that most of the compounds of the 5a–f series were found to be more potent than their corresponding isomers of the 7a–f series. The superiority of the former could be attributed to the higher basicity given by amines than imines as alkylated amines have yielded more fruitful results than alkylated imines. Interestingly, the replacement of the carbonyl group of the isatin ring with pyrrole to afford compound 5f produced stronger antifungal activity than for compound 5e. This illustrated that the pyrrole ring played a significant role in enhancing the activity of the targeted compounds in both series. Moreover, the chloro-substituted derivative 5b favored the activity rather than the fluoro-substituted derivative 5e.

**Table 5:** ED_50_ values for all the treated compounds for the plant pathogen *Rhizoctonia solani*

Compounds	ED_50_^a^ (μM)	Lower fiducial limit	Upper fiducial limit	*χ* ^2^	Regression equation
5a	2.47	1.82	3.349	0.165	0.96*x* ± 0.36
5b	**2.33**	**1.711**	**3.164**	**0.186**	**0.96*x* ± 0.36**
5c	2.87	2.259	3.689	0.247	1.28*x* ± 0.48
5d	**2.44**	**1.917**	**3.103**	**1.734**	**1.2*x*** ± **0.3**
5e	3.38	2.695	4.372	1.247	1.6*x* ± 0.6
5f	**0.96**	**0.597**	**1.319**	**2.735**	**1*x* ± 0.1**
6	2.50	1.992	3.147	2.299	1.12*x* ± 0.52
7a	2.56	1.89	3.502	0.212	0.96*x* ± 0.36
7b	2.75	1.955	3.951	0.718	0.8*x* ± 0.4
7c	2.65	1.964	3.636	0.251	0.96*x* ± 0.36
7d	2.51	1.801	3.528	0.341	0.96*x* ± 0.36
7e	2.77	2.208	3.528	1.35	1.6*x* ± 0.6
7f	**1.48**	**0.963**	**2.036**	**1.51**	**0.96*x* ± 0.16**
8	2.54	1.807	3.59	1.431	0.96*x* ± 0.36
**Hexaconazole 5% SC**	**2.44**	1.813	3.318	0.164	0.96*x* ± 0.36

a ED_50_ is the effective dose that causes 50% of the maximal cause.

### 2.3. Molecular docking

**Fig. 5:**
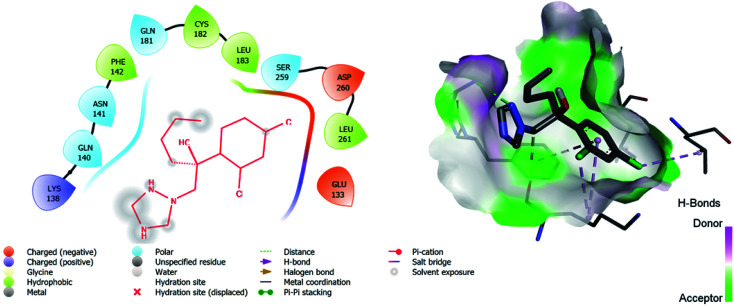
2D and 3D images of the standard drug (hexaconazole) with target protein CYP51.

**Fig. 6:**
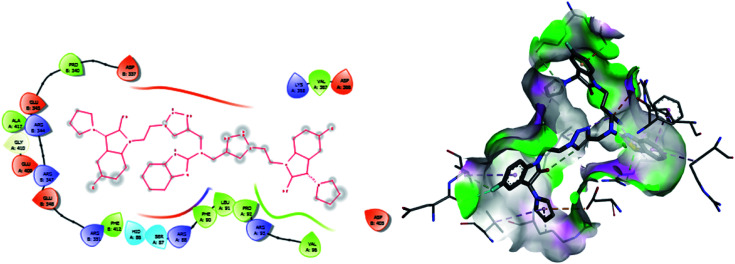
2D and 3D images of compound 5f with target protein CYP51.

**Fig. 7:**
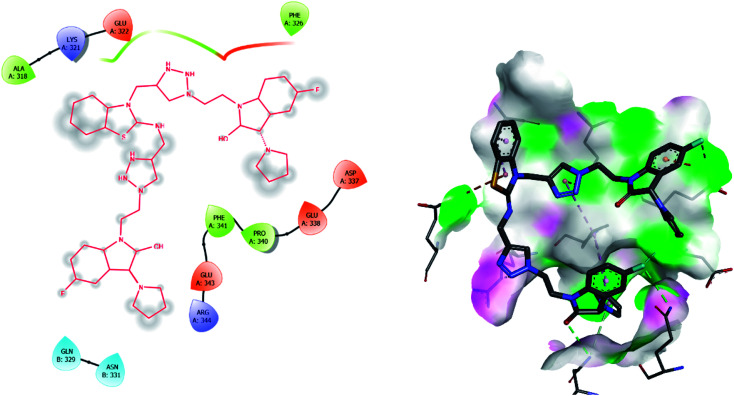
2D and 3D images of compound 7f with target protein CYP51.

**Fig. 8:**
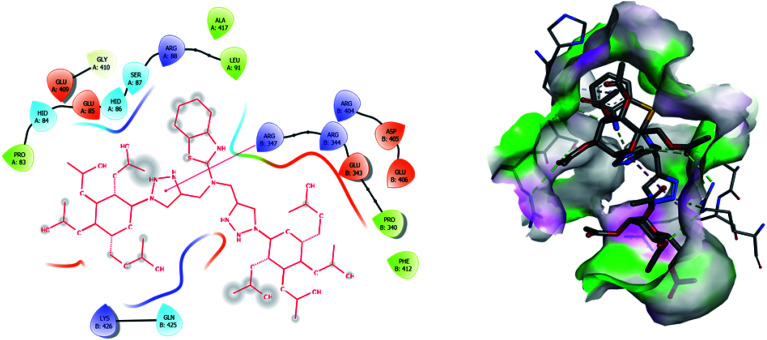
2D and 3D images of compound 6 with target protein CYP51.

**Fig. 9:**
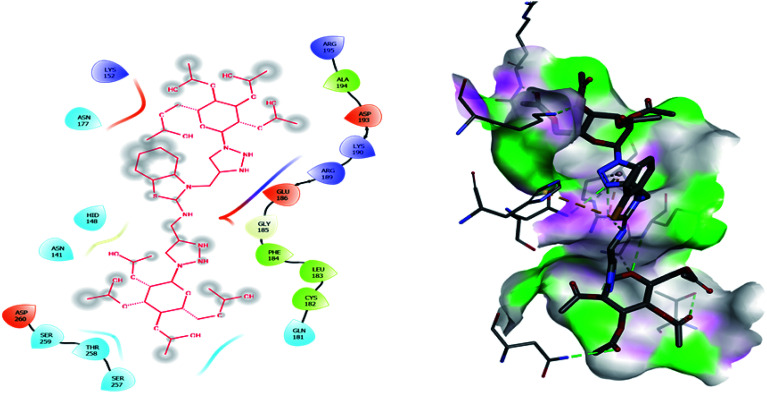
2D and 3D images of compound 8 with target protein CYP51.

Molecular docking has been developed as an exigent tool to determine the molecular targets for various ligands, mainly in the presence of minimum resources to execute enzymatic assays. Therefore, such tools have now become an essential part of drug discovery research. The 14α-demethylase enzyme is required for the biosynthesis of ergosterol, which is an essential component of the fungal cell membrane. Inhibition of CYP51 will prevent the synthesis of ergosterol, which will consequently diminish cell growth in fungi. To observe the putative binding mode of the compounds to the targeted protein, sterol 14α-demethylase enzyme (CYP51), (PDB entry: 3GW9) molecular docking studies have been performed with minor modifications by using AutoDock Vina.^24,25^ Deep analysis of the 2D and 3D poses of compounds 5f, 6, 7f, and 8 were performed in a similar manner to that of hexaconazole, the reference drug (Fig. 5–9). The hydrogen bonding between the donor and acceptor groups and targeted protein is shown in the 3D orientation, which can be observed in the 3D images. A similar binding mode with the hydrophobic and hydrophilic part of the ligand and the active site of CYP51 of some other compounds is given in Table 6.

**Table 6:** The interaction analysis of the molecular docking study on 14α-demethylase CYP51 with the benzothiazole-appended bis-triazole derivative-based structural isomers

S. N.	Drug name (ligand)	Binding energy (kcal mol^−1^)	Interaction with amino acids
1	6	−8.5	PRO A: 83, HIDA: 84, GLU A: 85, HID A: 86, SER A: 87, ARG A: 88, LEU A: 91, GLU A: 409, GLY A: 410, ALA A: 417
			ARG B: 347, ARG B: 344, GLU B: 343, PRO B: 340, PHE B: 412, ARG B: 405, ASP B: 405, GLU B: 406, LYS B: 426, GLN B: 425
2	8	−7.1	ASN: 141, HID: 148, ASN: 152, ASN: 177, GLN: 181, CYS: 182, LEU: 183, PHE: 184, GLY: 185, GLU: 186, ARG: 189, LYS: 190, ASP: 193, ALA: 194, ARG: 195, SER: 257, THR: 258, SER: 259, ASP: 260
3	5f	**−11.3**	HID A: 86, SER A: 87, PHE A: 90, LEU A: 91, PRO A: 92, ARG A: 93, VAL A: 96, ASP B: 337, PRO B: 340, GLU B: 343, ARG B: 344, ARG B: 347, GLU B: 348, ARG B: 351, ASP A: 366, VAL A: 367, LYS A: 368, ASP B: 405, GLU A: 409, GLY A: 410, ALA A: 417
4	7f	−8.4	ALA A: 318, LYS A: 321, GLU A: 322, PHE A: 326, ASP A: 337, GLU A: 338, PRO A: 340, PHE A: 341, GLU A: 343, ARG A: 344 GLN B: 329, ASN B: 331
5	**Hexaconazole**	**−6.2**	LYS: 138, GLN: 140, ASN: 141, PHE: 142, GLN: 181, CYS: 182, LEU: 183, SER: 259, ASP: 260, LEU: 261, GLU: 133

The binding interactions of the most potent antifungal compounds 5f and 7f were explained for their comparison. Both compounds have different orientations, which in turn results in different modes of binding in the active site of CYP51. In Fig. 6, it can be seen that the pyrrole side chains showed favorable steric interaction with the PRO B: 340, GLU B: 343, ARG B: 344, and ASP B: 405 residues and the side chain of the 1,2,3-triazole scaffolds was observed to be engaged in interaction with the PHE A: 90, LEU A: 91, and PRO A: 92 residues. In addition to these interactions, the benzothiazole core side-chain appeared to be involved in interactions with the HID A: 86, SER A: 87, and ARG A: 88 residues. As we speculated, compound 5f was found to fit very efficiently in the active site of the 14α-demethylase enzyme (CYP51). Fig. 6 illustrates the binding mode of compound 7f, where the pyrrole side chain shows interaction with the ASP A: 337, GLU A: 338, and ARG A: 344 residues, and the side chains of the 1,2,3-triazole scaffolds were observed to be engaged in interactions with the PHE A: 341 residue. Moreover, the benzothiazole core side-chain was observed to be engaged in interactions with the ALA A: 318, LYS A: 321, and GLU A: 322 residues.

From Table 6, it can be seen that the higher negative binding energy value of 5f (−11.3 kcal mol^−1^) compared to 7f (−8.4 kcal mol^−1^) indicated more favorable binding interactions with the CYP51 enzyme, which could explain the reason for the superiority of compound 5f over 7f. These docking results substantially agree with the above biological assay data and unveil the appropriate 14α-demethylase inhibitor design.

## 3. Conclusion

Two novel series (5a–f, 6 and 7a–f, 8) of benzothiazole-appended bis-triazole derivative-based fungicides that are structural isomers of each other were synthesized using click chemistry and were evaluated for their antifungal activity. The synthetic procedure reported here is reliable, simple, and highly reproducible. All the synthesized compounds showed excellent growth inhibition against *Rhizoctonia solani*. Moreover, the compounds 5f, 7f, and 5b inhibited the fungal growth better than the standard drug hexaconazole. Compound 5f was found to be approximately three times more effective than hexaconazole. Most of the compounds of the 5a–f series exhibited a higher antifungal inhibitory index than their corresponding isomers of the 7a–f series. Also, the replacement of the carbonyl group of the isatin ring with pyrrole was the paramount combination for promising antifungal activity to be focused on for medical applications in the era of fungal diseases. Moreover, molecular docking studies strengthened the results of the biological evaluation that predicted compound 5f as the most active with the highest binding energy (−11.3 kcal mol^−1^). Thus, the overall studies concluded that benzothiazole-appended bis-triazole derivatives could act as potent inhibitors of 14α-demethylase. Therefore, these compounds could be used as an alternative to the commercially available drug hexaconazole for the control of fungal growth in various plants.

## Conflicts of interest

The authors do not have any conflict of interest.

## Supplementary Material

RA-012-D2RA04465J-s001
