# Supplementary material for: Synthesis of benzothiazole-appended bis-triazole-based structural isomers with promising antifungal activity against Rhizoctonia solani
Source: RSC Adv. 2022 Aug 30;12(37):24412–26. doi: 10.1039/d2ra04465j (PMC9425831; doi:10.1039/d2ra04465j)
Supplement: RA-012-D2RA04465J-s001 [file RA-012-D2RA04465J-s001.pdf]

Electronic Supplementary Material (ESI) for RSC Advances.
This journal is © The Royal Society of Chemistry 2022

SUPPORTING INFORMATION

Synthesis of Benzothiazole-Appended Bis-triazoles based Structural Isomers with Promising Antifungal Activity against *Rhizoctonia solani*

Ravindra Kumar Upadhyay[a], Keshav Kumar Saini[a], Nidhi Deswal[a], Tejveer Singh[b], Kailash Pati Tripathi[c], Parshant Kaushik[c], Najam Akhtar Shakil[c], Alok Chandra Bharti[d], and Rakesh Kumar[a]*

[a] Department of Chemistry, University of Delhi, Delhi -110007 India.
[b] Molecular Oncology Laboratory, Department of Zoology, University of Delhi, and School of Life Sciences, JNU-Delhi, India.
[c] Division of Agricultural chemicals, Indian Agricultural Research Institute, New Delhi India.
[d] Molecular Oncology Laboratory, Department of Zoology, University of Delhi, Delhi -110007

CONTENT

1. **Synthesis of intermediates and target compounds** S1
2. **Characterization Data for 1, 2, 5a-e, 7a-e, 5f, 7f, 6 and 8.** S2
3. **^{1}H and ^{13}C Spectra of 1, 2, 5a-e, 7a-e, 5f, 7f, 6 and 8.** S7

General

All the solvents and reagents (analytical grade) were purchased from commercial suppliers and used for synthesis without further purification. Optimization of reactions was monitored using silica gel precoated thin layer chromatographic (TLC) plates. Melting points were determined on a Buchi instrument (M-560) and were uncorrected. Infra-red spectra were recorded using KBr disks on SHIMADZU IR Affinity 1S spectrophotometer. ^{1}H-NMR and ^{13}C-NMR spectra were recorded in deuterated DMSO-d_6 on JEOL, ECX-400P Spectrometer USA at 400 MHz and 100 MHz respectively. Chemical shifts, coupling constants, and absorption frequency values have been expressed in terms of δ (ppm), J (Hz), and ν (cm^{-1}), respectively. The abbreviations have been used in the spectral data as singlet (s), doublet (d), triplet (t), quartet (q), doublet of doublet (dd), and multiplet (m). Mass spectrometry measurements were obtained on 6530 Accurate-Mass Q-TOF LC/MS spectrometer. The fungus *Rhizoctonia solani* was self-cultured at the appropriate environmental condition in the lab and the media PDA potato dextrose agar were purchased from Spectrochem.

A general method for the synthesis of bis-propargylated 2-aminobenzothiazoles 1 and 2. The reaction was carried out by using 1 mmol of benzothiazole and 2 mmol of Propargyl bromide and reaction mixture was heated in presence of mild base K_2CO_3 and acetonitrile as solvent at 70$^{\circ C}$ for 8hrs. At this condition

50% yield of the both products are formed. Both the products **1** and **2** were separated via silica gel chromatography (100-200 mesh size) by using hexane and ethylacetate (98:2) as a solvent system.

A general method for the synthesis of compounds 5a-e and 7a-e. To a stirred solution of compounds **1 or 2** (1 mmol) and **3a-e** (2 mmol) in DMF: H_2O (1:1), copper sulphate (0.4 mmol) and sodium ascorbate (0.9 mmol) were added and Heat for 20 mins at 80ºC. The completion of the reaction was checked by TLC. The resulting reaction mixture was poured onto 30 mL ice-water and then extracted with chloroform (3 × 30 mL). The combined extracts were washed with brine solution, dried over anhydrous sodium sulphate, filtered and then concentrated at reduced pressure under vacuum resulting in the isolation of the crude product. The crude product was further purified via silica gel chromatography (100-200 mesh size) to give desired hybrids **5a-e, and 7a-e,** in 70-90% yields.

A general method for the synthesis of compounds 5f and 7f.

Compound **5e** or **7e** in round bottom flask, (1 mmol) were refluxed with L-hydroxyproline (2.3 mmol) in presence of $InCl_3$ as Lewis acid catalyst in ethanol for 2-3 hrs. The completion of the reaction was checked by TLC. The resulting reaction mixture solvent was evaporated at rotavapour, then extracted with chloroform (3 × 30 mL). The combined extracts were washed with brine solution, dried over anhydrous sodium sulphate, filtered and then concentrated at reduced pressure under vacuum resulting in the isolation of the crude product. The crude product was further purified via silica gel chromatography (100-200 mesh size) to give desired hybrids (**5f and 7f**).

A general method for the synthesis of compounds 6 and 8.

To a stirred solution of compounds **1 or 2** (1 mmol) and **4** (2 mmol) in DMF: H_2O (1:1), copper sulphate (0.4 mmol) and sodium ascorbate (0.9 mmol) were added and Heat for 15-25 mins at 75-80ºC. The completion of the reaction was checked by TLC. The resulting reaction mixture was poured onto 30 mL ice-water and then extracted with chloroform (3 × 30 mL). The combined extracts were washed with brine solution, dried over anhydrous sodium sulphate, filtered and then concentrated at reduced pressure under vacuum resulting in the isolation of the crude product. The crude product was further purified via silica gel chromatography (100-200 mesh size) to give desired hybrids **(6 and 8),** in 85% and 80%, respectively yields.

1,1'-(2,2'-(5,5'-(benzo[d]thiazol-2-ylazanediyl) bis(methylene)bis(1H-1,2,3-triazole-5,1-diyl)) bis(ethane-2,1-diyl)) diindoline-2,3-dione (5a). Orange Solid; Yield: 78%; mp:154-156 ˚C; IR (KBr, cm⁻

^{1}) 1734, 1606, 1531, 752, 474; ^{1}H NMR (400 MHz, DMSO-d_6): δ 8.14 (s, 2H), 7.69 (d, J = 8.7 Hz, 1H), 7.43 – 7.38 (m, 5H), 7.26 – 7.21 (m, 1H), 7.03 (t, J = 8.2 Hz, 1H), 6.94 (t, J = 7.5 Hz, 2H), 6.74 (d, J = 8.1 Hz, 2H), 4.59 (t, J = 5.6 Hz, 4H), 4.47 (s, 4H), 4.06 (t, J = 5.6 Hz, 4H); ^{13}C NMR (100 MHz, DMSO-d_6): δ 183.42, 167.71, 158.65, 152.87, 150.69, 142.79, 138.48, 131.23, 126.41, 125.13, 124.97, 123.76, 121.56, 117.79, 110.53, 47.59, 44.92; MS data: calcd mass (M+H)$^+$, 659.1, found, 659.1.

1,1'-(2,2'-(5,5'-(benzo[d]thiazol-2-ylazanediyl) bis(methylene)bis(1H-1,2,3-triazole-5,1-diyl)) bis(ethane-2,1-diyl)) bis(5-chloroindoline-2,3-dione) (5b). Orange Solid; Yield: 80%; mp:156-158 ˚C; IR (KBr, cm^{-1}) 1738, 1607, 1540, 747, 469; ^{1}H NMR (400 MHz, DMSO- d_6): δ 8.17 (s, 2H), 7.72 (d, J = 8.5 Hz, 1H), 7.53 – 7.39 (m, 5H), 7.29 – 7.22 (m, 1H), 7.06 (t, J = 7.1 Hz, 1H), 6.78 (d, J = 9.2 Hz, 2H), 4.59 (t, J = 5.6 Hz, 4H), 4.51 (s, 4H), 4.08 (t, J = 5.5 Hz, 4H); ^{13}C NMR (100 MHz, DMSO-d_6): δ 184.65, 164.03, 157.59, 151.97, 148.84, 142.16, 137.10, 136.13, 134.55, 128.88, 128.75, 127.18, 125.87, 122.67, 122.47, 119.52, 112.56, 110.27, 49.06, 45.72; MS data: calcd mass (M+H)$^+$, 727.1, found, 727.0.

1,1'-(2,2'-(5,5'-(benzo[d]thiazol-2-ylazanediyl) bis(methylene)bis (1H-1,2,3-triazole-5,1-diyl)) bis(ethane-2,1-diyl)) bis(5-bromoindoline-2,3-dione) (5c). Orange Solid; Yield: 90%; mp: 161-164 ˚C C; IR (KBr, cm^{-1}) 1737, 1604, 1533, 748, 478; ^{1}H NMR (400 MHz, DMSO- d6): δ 8.19 (s, 2H), 7.75 (d, J = 7.7 Hz, 1H), 7.67 – 7.59 (m, 3H), 7.47 (d, J = 7.9 Hz, 2H), 7.29 (t, J = 7.6 Hz, 1H), 7.09 (t, J = 7.4 Hz, 1H), 6.77 (d, J = 8.4 Hz, 2H), 4.65 – 4.60 (m, 4H), 4.55 (s, 4H), 4.11 (t, J = 5.4 Hz, 4H); ^{13}C NMR (100 MHz, DMSO-d_6): δ 182.13, 167.64, 158.22, 152.82, 149.56, 142.73, 140.14, 131.14, 127.16, 126.37, 125.20, 121.71, 121.54, 119.51, 119.11, 115.53, 112.68, 47.48, 44.88; MS data: calcd mass (M+H)$^+$, 815.0, found, 815.8.

1,1'-((((benzo[d]thiazol-2-ylazanediyl) bis(methylene)) bis(1H-1,2,3-triazole-5,1-diyl)) bis(ethane-2,1-diyl)) bis(5-methylindoline-2,3-dione) (5d). Orange Solid; Yield: 89%; mp: 185-187˚C; IR (KBr, cm^{-1}); 1739, 1618, 1484, 762, 478; ^{1}H NMR (400 MHz, DMSO- d_6): δ 8.16 (s), 7.44 (d, J = 0.7 Hz), 7.42 (d, J = 0.5 Hz), 7.27 (d, J = 1.3 Hz), 7.25 – 7.24 (m), 7.23 (d, J = 1.3 Hz), 7.22 – 7.21 (m), 7.20 – 7.19 (m), 7.17 (dd, J = 1.7, 0.6 Hz), 7.08 – 7.03 (m), 6.60 (d, J = 8.1 Hz), 4.61 – 4.56 (m), 4.46 (s), 4.04 (t, J = 5.6 Hz), 2.12 (s); ^{13}C NMR (100 MHz, DMSO-d_6): δ 183.62, 167.70, 158.71, 152.80, 148.58 142.79, 138.79, 133.16, 131.17, 126.43, 125.17, 121.68, 119.08, 117.74, 110.35, 47.66, 44.92 , 20.45 (s); MS data: calcd mass (M+H)$^+$, 687.2, found, 687.7.

1,1'-((((benzo[d]thiazol-2-ylazanediyl) bis(methylene)) bis (1H-1,2,3-triazole 5,1diyl)) bis(ethane-2,1-diyl)) bis(5-fluoroindoline-2,3-dione) (5e). Orange Solid; Yield: 90%; mp:171-172 ˚C; IR (KBr, cm^{-1});

1742, 1618, 1470, 797, 491. ^{1}H NMR (400 MHz, DMSO- d_6): δ 8.20 (s, 2H), 7.74 (d, *J* = 7.8 Hz, 1H), 7.46 (d, *J* = 8.0 Hz, 1H), 7.36 (dd, *J* = 13.5, 5.4 Hz, 4H), 7.28 (t, *J* = 8.0 Hz, 1H), 7.09 (t, *J* = 7.6 Hz, 1H), 6.83 (d, *J* = 9.4 Hz, 2H), 4.63 (t, *J* = 5.6 Hz, 4H), 4.57 (s, 4H), 4.11 (t, *J* = 5.5 Hz, 4H); ^{13}C NMR (100 MHz, DMSO-d_6): δ 182.75, 167.62, 160.05, 158.63, 157.65, 152.81, 146.88, 142.75, 131.13, 126.34, 125.16, 124.55, 124.31, 121.69, 121.49, 119.08, 118.69, 118.62, 112.05, 111.97, 111.73, 47.46, 44.95; MS Data: calcd mass $(M+H)^+$, 695.1, found, 695.2.

(E)-1-(2-(4-((3-((1-(2-(2,3-dioxoindolin-1-yl) ethyl)-1H-1,2,3-triazol-4-yl) methyl) benzo[d]thiazol-2(3H)-pyridinamine) methyl)-1H-1,2,3-triazol-1-yl) ethyl) indoline-2,3-dione (7a). Orange Solid; Yield: 70%; mp:165-168 ˚C; IR (KBr, cm^{-1} 1735, 1611, 1474, 755, 475).; ^{1}H NMR (400 MHz, DMSO-d_6): *δ* 8.04 (d, *J* = 1.5 Hz), 7.52 – 7.41 (m), 7.28 (td, *J* = 7.8, 1.3 Hz), 7.23 – 7.18 (m), 7.11 (d, *J* = 7.8 Hz), 7.01 – 6.91 (m), 6.87 (d, *J* = 8.0 Hz), 6.69 (d, *J* = 8.0 Hz), 5.09 (s), 4.57 (dt, *J* = 11.5, 5.8 Hz), 4.27 (s), 4.06 (dt, *J* = 19.1, 5.8 Hz); ^{13}C NMR (100 MHz, DMSO-d_6): *δ* 183.48 (s), 158.62 (d, *J* = 3.2 Hz), 150.71 (s), 146.64 (s), 142.80 (s), 140.15 (s), 138.63 (s), 138.41 (s), 127.04 (s), 125.70 – 124.36 (m), 124.01 (s), 124.00 – 123.49 (m), 122.89 (s), 121.74 (d, *J* = 14.6 Hz), 117.78 (d, *J* = 12.8 Hz), 110.85 (s), 110.55 (s), 49.70(s), 47.40 (d, *J* = 19.9 Hz) 38.59 (s); MS data: calcd mass $(M+H)^+$, 659.1, found, 659.1.

(E)-5-chloro-1-(2-(4-((3-((1-(2-(5-chloro-2,3-dioxoindolin-1-yl) ethyl)-1H-1,2,3-triazol-4-yl) methyl) benzo[d]thiazol-2(3H)-ylideneamino) methyl)-1H-1,2,3-triazol-1-yl) ethyl) indoline-2,3-dione (7b). Orange Solid; Yield: 75%; mp:175-177 ˚C; IR (KBr, cm^{-1}) 1745, 1605, 1475, 739, 459, 474.; ^{1}H NMR (400 MHz, DMSO-d_6): *δ* 8.08 (d, *J* = 5.9 Hz, 2H), 7.59 (d, *J* = 10.6 Hz, 1H), 7.54 (dd, *J* = 7.5, 2.2 Hz, 3H), 7.32 (d, *J* = 8.5 Hz, 1H), 7.24 (t, *J* = 7.4 Hz, 1H), 7.15 (d, *J* = 8.0 Hz, 1H), 7.02 (t, *J* = 7.5 Hz, 1H), 6.93 (d, *J* = 8.5 Hz, 1H), 6.70 (d, *J* = 8.5 Hz, 1H), 5.14 (s, 2H), 4.63 – 4.57 (m, 4H), 4.32 (s, 2H), 4.10 (dt, *J* = 20.9, 5.5 Hz, 4H); ^{13}C NMR (100 MHz, DMSO-d_6): *δ* 182.34 (s), 158.33 (s), 156.10 (s), 149.19 (s), 146.61 (s), 142.79 (s), 140.04 (s), 137.45 (s), 137.21 (s), 128.02 (d, *J* = 8.4 Hz), 126.94 (s), 124.83 (s), 124.43 (s), 123.96 (s), 122.87 (s), 121.66 (d, *J* = 9.1 Hz),119.06 (d, *J* = 10.9 Hz), 112.56 (s), 112.20 (s), 110.23 (s), 49.63 (s), 47.47 (s), 47.21 (s), 38.53 (s); MS data: calcd mass $(M+H)^+$, 727.1, found, 727.0.

(E)-5-bromo-1-(2-(4-((3-((1-(2-(5-bromo-2,3-dioxoindolin-1-yl) ethyl)-1H-1,2,3-triazol-4-yl) methyl) benzo[d]thiazol-2(3H)-ylideneamino) methyl)-1H-1,2,3-triazol-1-yl) ethyl) indoline-2,3-dione (7c). Orange Solid; Yield: 80%; mp:168-171 ˚C; IR (KBr, cm^{-1}) 1739, 1621, 1529, 749, 466; ^{1}H NMR (400 MHz, DMSO-d_6): *δ* 8.04 (d, *J* = 6.6 Hz), 7.68 (d, *J* = 9.8 Hz), 7.60 (d, *J* = 10.1 Hz), 7.50 (d, *J* = 7.5 Hz), 7.42 (d, *J* = 8.5 Hz), 7.20 (t, *J* = 7.6 Hz), 7.11 (d, *J* = 7.9 Hz), 6.98 (t, *J* = 7.5 Hz), 6.84 (d, *J* = 8.5 Hz),

6.62 (d, J = 8.4 Hz), 5.11 (s), 4.53 (dd, J = 13.9, 8.8 Hz), 4.27 (s), 4.05 (dt, J = 19.6, 5.0 Hz); ^{13}C NMR (100 MHz, DMSO-d$_6$): δ 182.25 (s), 158.24 (s), 156.19 (s), 149.60 (s), 146.65 (s), 142.83 (s), 141.59 – 139.10 (m), 127.14 (d, J = 24.6 Hz), 126.89 – 126.40 (m), 124.88 (s), 124.03 (s), 122.96 (s), 121.70 (d, J = 7.9 Hz), 119.53 (d, J = 10.4 Hz), 113.86 – 113.45 (m), 112.86 (d, J = 35.6 Hz), 110.30 (s), 49.71 (s), 47.52 (s), 47.25 (s) 38.56 (s); MS Data: calcd mass (M+H)$^+$, 815.0, found, 815.8.

(Z)-5-methyl-1-(2-(4-(((3-((1-(2-(5-methyl-2,3-dioxoindolin-1-yl) ethyl)-1H-1,2,3-triazol-4-yl) methyl) benzo[d]thiazol-2(3H)-ylidene) amino) methyl)-1H-1,2,3-triazol-1-yl) ethyl) indoline-2,3-dione (7d). Orange Solid; Yield: 75%; mp:167-169 ˚C; IR (KBr, cm^{-1}) 1742, 1616, 1478, 750, 478; ^{1}H NMR (400 MHz, DMSO-d$_6$): δ 8.05 (d, J = 5.4 Hz), 7.51 (d, J = 7.6 Hz), 7.30 – 7.19 (m), 7.14 (d, J = 8.0 Hz), 7.08 – 6.96 (m), 6.79 (d, J = 8.0 Hz), 6.54 (d, J = 8.1 Hz), 5.10 (s), 4.55 (dt, J = 11.5, 5.7 Hz), 4.28 (s), 4.05 (t, J = 5.7 Hz), 4.00 (t, J = 5.7 Hz), 2.14 (s), 2.12 (s); ^{13}C NMR (100 MHz, DMSO-d$_6$): δ 183.63 (d, J = 5.5 Hz), 158.65 (d, J = 2.3 Hz), 148.55 (s), 140.12 (s), 138.91 (s), 138.71 (s), 133.16 (d, J = 13.9 Hz), 127.05 (s), 125.19 (d, J = 5.7 Hz), 124.86 (s), 123.91 (s), 122.93 (s), 121.78 (s), 117.73 (d, J = 12.1 Hz), 110.76 (s), 110.40 (s), 49.63 (s), 47.56 (s), 49.70 – 40.61 (m), 20.50 (s); MS data: calcd mass (M+H)$^+$, 687.2, found, 687.7.

(Z)-5-fluoro-1-(2-(4-(((3-((1-(2-(5-fluoro-2,3-dioxoindolin-1-yl) ethyl)-1H-1,2,3-triazol-4-yl) methyl) benzo[d]thiazol-2(3H)-ylidene) amino) methyl)-1H-1,2,3-triazol-1-yl) ethyl) indoline-2,3-dione (7e). Orange Solid; Yield: 76%; mp:175-177 ˚C; IR (KBr, cm^{-1}) 1739, 1626, 1488, 796, 432; ^{1}H NMR (400 MHz, DMSO-d$_6$): δ 8.08 (d, J = 2.9 Hz, 2H), 7.53 (d, J = 7.6 Hz, 1H), 7.41 (dt, J = 12.7, 9.3 Hz, 3H), 7.24 (t, J = 7.5 Hz, 1H), 7.19 – 7.12 (m, 2H), 7.02 (t, J = 7.5 Hz, 1H), 6.95 (dd, J = 8.5, 3.6 Hz, 1H), 6.72 (dd, J = 8.7, 3.6 Hz, 1H), 5.14 (s, 2H), 4.63 – 4.57 (m, 4H), 4.31 (s, 2H), 4.10 (dt, J = 20.7, 5.5 Hz, 4H); ^{13}C NMR (100 MHz, DMSO-d$_6$): δ 182.82 (s), 160.03 (d, J = 14.9 Hz), 158.62 (s), 157.63 (d, J = 15.2 Hz), 156.09 (s), 146.85 (s), 146.58 (s), 142.76 (s), 140.04 (s), 126.95 (s), 124.87 (s), 124.64 (s), 124.40 (s), 124.05 (d, J = 16.5 Hz), 122.85 (s), 121.64 (d, J = 6.4 Hz), 118.71 (s), 112.32 (s), 111.93 (d, J = 12.9 Hz), 110.25 (s), 49.60 (s), 47.98 – 47.46 (m), 47.31 (d, J = 26.5 Hz), 38.48 (s); MS Data: calcd mass (M+H)$^+$, 695.1, found, 695.2.

1,1'-((((benzo[d]thiazol-2-ylazanediyl)bis(methylene))bis(1H-1,2,3-triazole-5,1-diyl))bis(ethane-2,1-diyl))bis(5-fluoro-3-(1H-pyrrol-1-yl)indolin-2-one) (5f). white colour; Yield: 70%; mp: 171-172 ˚C; IR (KBr, cm^{-1}); 1605, 1533, 1411, 747, 433; ^{1}H NMR (400 MHz, DMSO- d$_6$): δ 8.12 (s, 2H), 7.75 (d, J = 7.6 Hz, 1H), 7.49 (d, J = 7.8 Hz, 1H), 7.29 (d, J = 4.1 Hz, 1H), 7.11 – 7.01 (m, 4H), 6.88 (d, J = 8.3 Hz, 4H), 6.64 (s, 4H), 6.03 (d, J = 1.5 Hz, 4H), 5.85 (s, 2H), 4.66 (t, J = 5.8 Hz, 4H), 4.55 (s, 4H), 4.19 –

4.12 (m, 4H); ^{13}C NMR (100 MHz, DMSO-d_6): δ 172.67 (s), 167.70 (s), 157.71 (s), 142.78 (s), 141.56 (s), 139.04 (s), 131.17 (s), 126.19 (s), 124.83 (s), 123.20 (s), 122.91 (s), 120.99 (d, *J* = 4.7 Hz), 114.46 (d, *J* = 22.1 Hz), 111.39 (dd, *J* = 19.2, 11.8 Hz), 110.27 (d, *J* = 6.7 Hz), 109.14 (d, *J* = 6.2 Hz), 60.04 (d, *J* = 3.1 Hz), 49.25 (s), 47.25 (s); MS Data: calcd mass $(M+H)^+$, 797.2, found, 797.2.

(Z)-5-fluoro-1-(2-(4-(((3-((1-(2-(5-fluoro-2-oxo-3-(1H-pyrrol-1-yl) indolin-1-yl) ethyl)-1H-1,2,3-triazol-4-yl) methyl) benzo[d]thiazol-2(3H)-ylidene) amino) methyl)-1H-1,2,3-triazol-1-yl) ethyl)-3-(1H-pyrrol-1-yl) indolin-2-one (7f). White Solid; Yield: 75%; mp:174-175 ˚C; IR (KBr, cm^{-1}) ; 1611, 1523, 1431, 755, 439; ^{1}H NMR (400 MHz, DMSO-d_6): δ 8.03 (d, *J* = 7.5 Hz, 2H), 7.54 (d, *J* = 7.7 Hz, 2H), 7.22 (d, *J* = 7.9 Hz, 2H), 7.12 (dd, *J* = 18.5, 7.5 Hz, 3H), 7.01 (d, *J* = 8.0 Hz, 1H), 6.91 (d, *J* = 8.8 Hz, 2H), 6.83 (t, *J* = 10.1 Hz, 1H), 6.65 (dd, *J* = 4.2, 2.0 Hz, 4H), 6.04 (d, *J* = 6.1 Hz, 4H), 5.91 (s, 1H), 5.85 (s, 1H), 5.15 (d, *J* = 3.4 Hz, 2H), 4.66 – 4.57 (m, 4H), 4.33 (s, 2H), 4.13 (dd, *J* = 26.4, 10.8 Hz, 4H); ^{13}C NMR (100 MHz, DMSO-d_6): δ 172.68 (s), 166.08(s), 159.08(s), 146.70 (s), 142.84 (s), 140.05 (s), 139.07 (s), 126.97 (s), 124.50 (s), 123.64 (s), 122.87 (s), 121.71 (s), 120.99 (d, *J* = 4.8 Hz), 116.79 – 116.37 (m), 116.23 (d, *J* = 22.7 Hz), 112.73 (s), 112.48 (s), 110.54 (d, *J* = 5.7 Hz), 110.12 (dd, *J* = 19.2, 11.8 Hz), 109.16 (s), 60.02 (d, *J* = 7.7 Hz), 49.63 (s), 47.27 (s), 46.98 (s), 38.54 (s); MS Data: calcd mass $(M+H)^+$, 797.2, found, 797.2.

(2R,5R,6S)-2-(acetoxymethyl)-6-(4-((benzo[d]thiazol-2-yl((1-((2R,3S,4R,6S)-3,4,5 triacetoxy-6-(acetoxymethyl) tetrahydro-2H-pyran-2-yl)-1H-1,2,3-triazol-4-yl) methyl) amino) methyl)-1H-1,2,3-triazol-1-yl) tetrahydro-2H-pyran-3,4,5-triyl triacetate (6). White Solid; Yield: 85%; mp: 193 -197 ˚C; IR (KBr, cm^{-1}) 1743, 1537, 1440, 1371, 1217, 1037; ^{1}H NMR (400 MHz, DMSO- d_6): δ 8.47 (s, 2H), 7.79 – 7.68 (m, 1H), 7.54 – 7.43 (m, 1H), 7.28 (dd, *J* = 13.4, 6.7 Hz, 1H), 7.08 (dd, *J* = 13.4, 6.8 Hz, 1H), 6.34 (dd, *J* = 8.7, 5.9 Hz, 2H), 5.65 (dd, *J* = 16.5, 7.5 Hz, 2H), 5.54 (dd, *J* = 15.2, 9.3 Hz, 2H), 5.16 (dd, *J* = 15.4, 9.6 Hz, 2H), 4.79 (s, 4H), 4.35 (d, *J* = 5.9 Hz, 2H), 4.11 (dd, *J* = 12.4, 6.9 Hz, 4H), 2.07 – 1.90 (m, 19H), 1.73 (d, *J* = 5.7 Hz, 6H);^{13}C NMR (100 MHz, DMSO-d_6): *δ* 170.47, 170.00, 169.81, 168.95, 167.71, 152.70, 143.52, 131.25, 126.37, 123.38, 121.80, 121.54, 119.20, 84.32, 73.75, 72.51, 70.66, 68.00, 62.23, 45.33, 20.94, 20.84, 20.69, 20.27; MS Data: calcd mass $(M+H)^+$, 973.2, found, 973.8.

(2R,3R,4S,5R)-2-(acetoxymethyl)-6-(4-((((Z)-3-((1-((3R,4S,5R,6R)-3,4,5-triacetoxy-6-(acetoxymethyl) tetrahydro-2H-pyran-2-yl)-1H-1,2,3-triazol-4-yl) methyl) benzo[d]thiazol-2(3H)-ylidene) amino) methyl)-1H-1,2,3-triazol-1-yl) tetrahydro-2H-pyran-3,4,5-triyl triacetate (8). White

Solid; Yield: 80%; mp:197-199 ˚C; IR (KBr, cm^{-1}) 1747, 1565, 1490, 1340, 1235, 1040; ^{1}H NMR (400 MHz, DMSO-d_6): δ 8.31 (d, *J* = 13.6 Hz), 7.52 (d, *J* = 7.6 Hz), 7.20 (t, *J* = 7.7 Hz), 7.11 (d, *J* = 8.2 Hz), 6.98 (t, *J* = 7.6 Hz), 6.27 (dd, *J* = 13.1, 9.2 Hz), 5.70 – 5.41 (m), 5.26 (s), 5.12 (td, *J* = 9.8, 6.4 Hz), 4.38 (s), 4.28 (s), 4.04 (t, *J* = 9.2 Hz), 2.01 – 1.83 (m), 1.75 (s), 1.62 (s); ^{13}C NMR (100 MHz, DMSO-d_6): δ 170.55 (d, *J* = 2.8 Hz), 169.99 (dd, *J* = 19.4, 3.9 Hz), 169.06 (s), 168.85 (s), 156.55 (s), 147.44 (s), 143.45 (s), 140.07 (s), 127.04 (s), 123.06 (d, *J* = 19.0 Hz), 122.34 (s), 121.81 (d, *J* = 6.0 Hz), 84.28 (d, *J* = 3.8 Hz), 73.76 (d, *J* = 12.3 Hz), 72.69 (d, *J* = 13.5 Hz), 70.53 (d, *J* = 11.2 Hz), 68.05 (s), 62.34 (s), 21.10 – 20.61 (m), 20.46 (s), 20.25 (s); MS Data: calcd mass $(M+H)^+$, 973.2, found, 973.8.

4.5. Experimental Protocol for Biological Activity

4.5.1. *Antifungal activity.*

Test Fungus

The test fungus *Rhizoctonia solani* was procured from Indian Type Culture Collection (ITCC-7479) center, Division of Plant Pathology, ICAR-Indian Agricultural Research Institute, NewDelhi-110012, and India and kept at 27°C for at least 4-7 days on Potato Dextrose Agar (PDA) slant. The fungus was sub cultured in Petri plates for further bioassay studies.

In vitro fungicidal activity: *In vitro* antifungal activity of the all-synthesized compounds was carried out against *Rhizoctonia solani* in PDA medium by using poisoned food technique.[27] Fungal growth in presence of compounds **5a-f, 6** and **7a-f, 8** was evaluated at five concentrations (0.62ppm, 1.25ppm, 2.50ppm, 5.00ppm, 10.00ppm were prepared from stock solution by serial dilution). The commercial fungicides, Hexaconazole 5% SC were taken as positive control.

Fungal growth (colony diameter) was measured and Percentage inhibition was calculated by Abbott's formula (Abbott, 1925).

Percentage inhibition(I) = (C−T) ×100/C,

Where C=colony diameter (mm) of the control and T=colony diameter (mm) of the test plate

Corrected % inhibition (IC) was calculated by the given formula

IC= (I−CF)/(100−CF) ×100

Where, I=Percentage inhibition, CF=(90-C)/C×100, 90 is the diameter (mm) of the petriplateand C is the growth of the fungus(mm) in control. ED_{50} (ppm) values (Effective Dose for 50% inhibition) were calculated using SPSS statistical Package (v16.0).

4.6. Molecular Docking.

***In silico* molecular docking:**

Docking was performed on dell Workstation with 2.50GHz processor, 8 GB RAM, 512 SSD i5 6th generation running in Windows 10 operating system. Receptor protein (3GW9) used from RCSB data bank.

Preparation of ligands: Ligands structure is drawn on Chemdraw software. The output file was .sdf format. Further these ligands files were converted to .pdb format using Online SMILES Translator.

Blind molecular docking: Molecular docking was performed as described earlier[28] with minor modifications. Receptor (3GW9) and ligand (benzothiazole-appended bis- triazole derivatives **5f**, **7f**, and **6**, **8**) molecules were performed docking by using AutoDock Vina. Center_x = 14.5, center_y = 23.694, center_z = 28.045 exhaustiveness = 8A, grid of 126, 126, and 126 points in the x, y, and z directions and the target molecules was centered by running the Autodock vina. The resultant log file was used for binding energy. The macromolecule and ligand complex were saved in .pdb format to be analysed in LigPlot. The visualization of structure files was done using the graphical interface of the LigPlot molecular graphics system and Discovery Studio Visualizer.

Spectra:

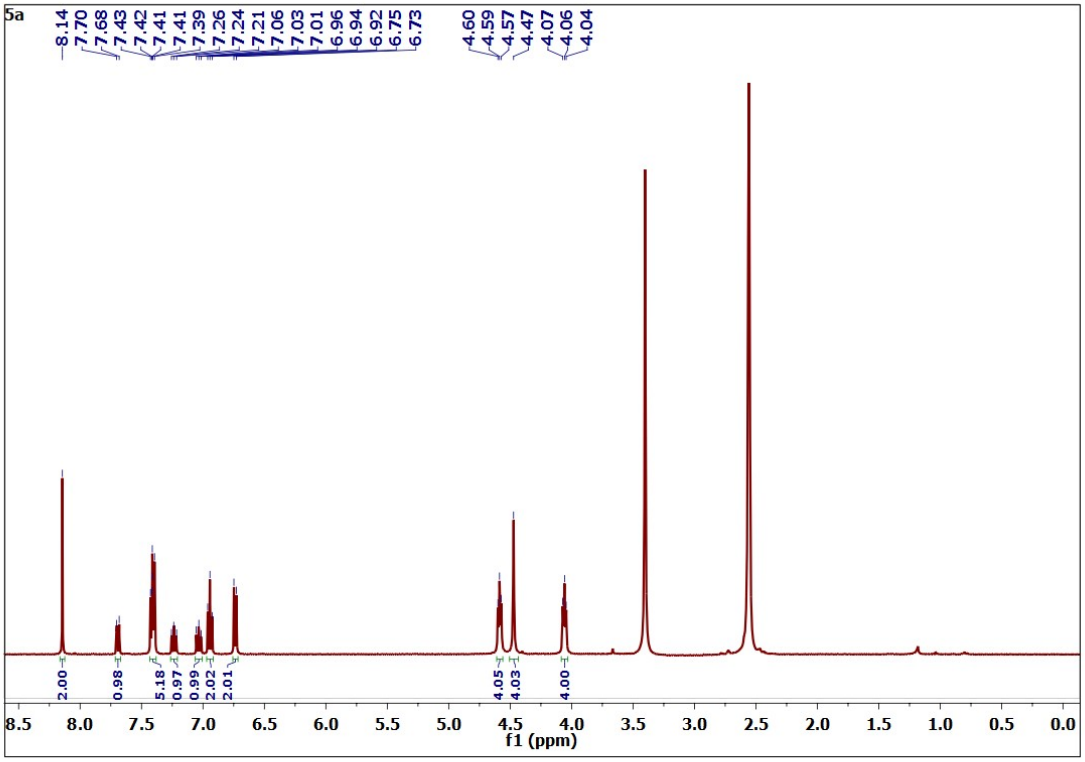

Figure S1. ^{1}H NMR spectrum of compound **5a** (400 MHz, DMSO-d_6).

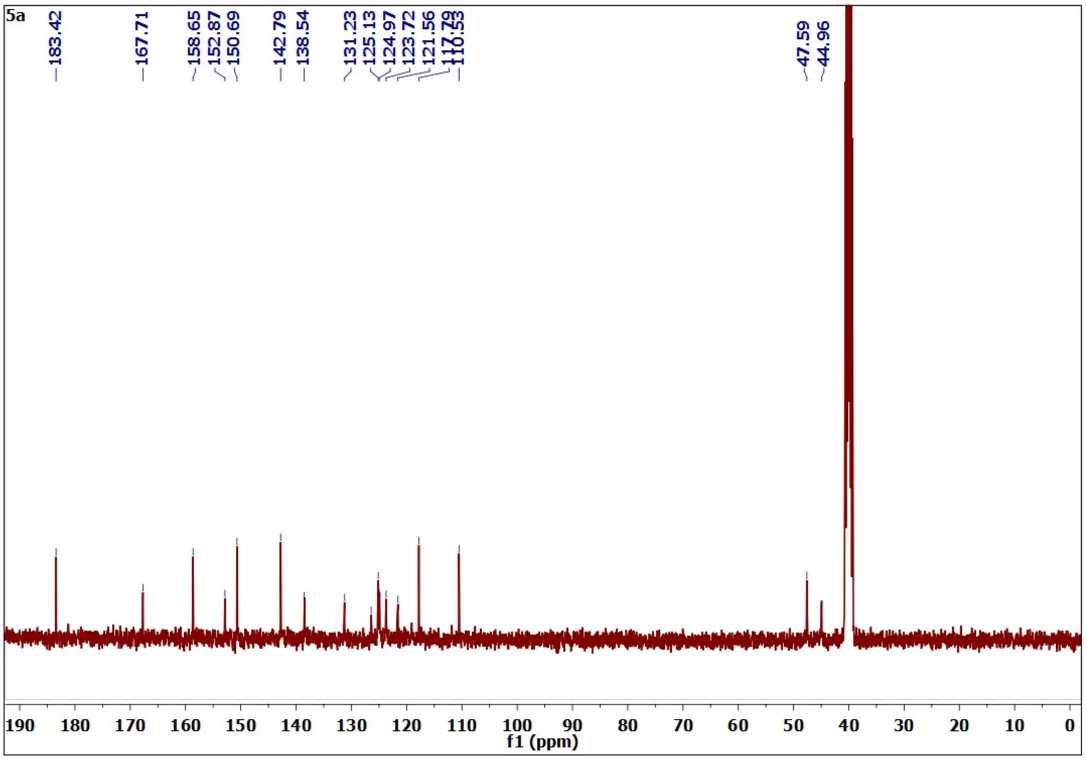

Figure S2. ^{13}C NMR spectrum of compound **5a** (100.6 MHz, DMSO-d_6)

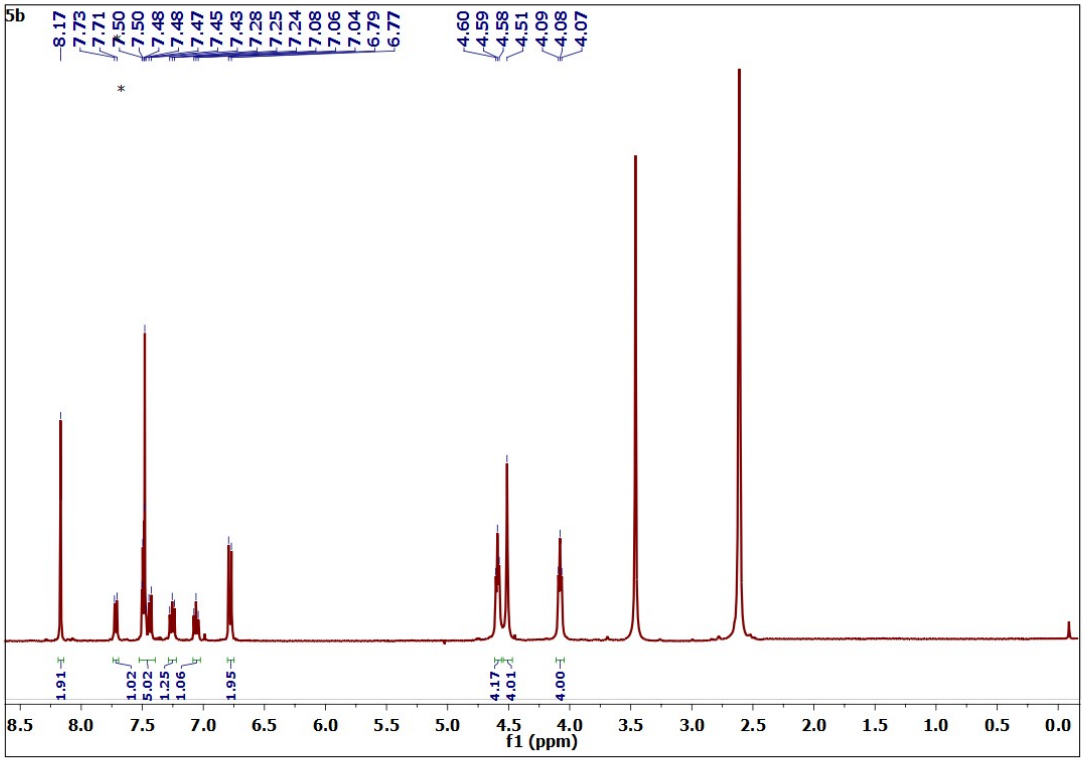

Figure S3. ^{1}H NMR spectrum of compound **5b** (400 MHz, DMSO-d_6).

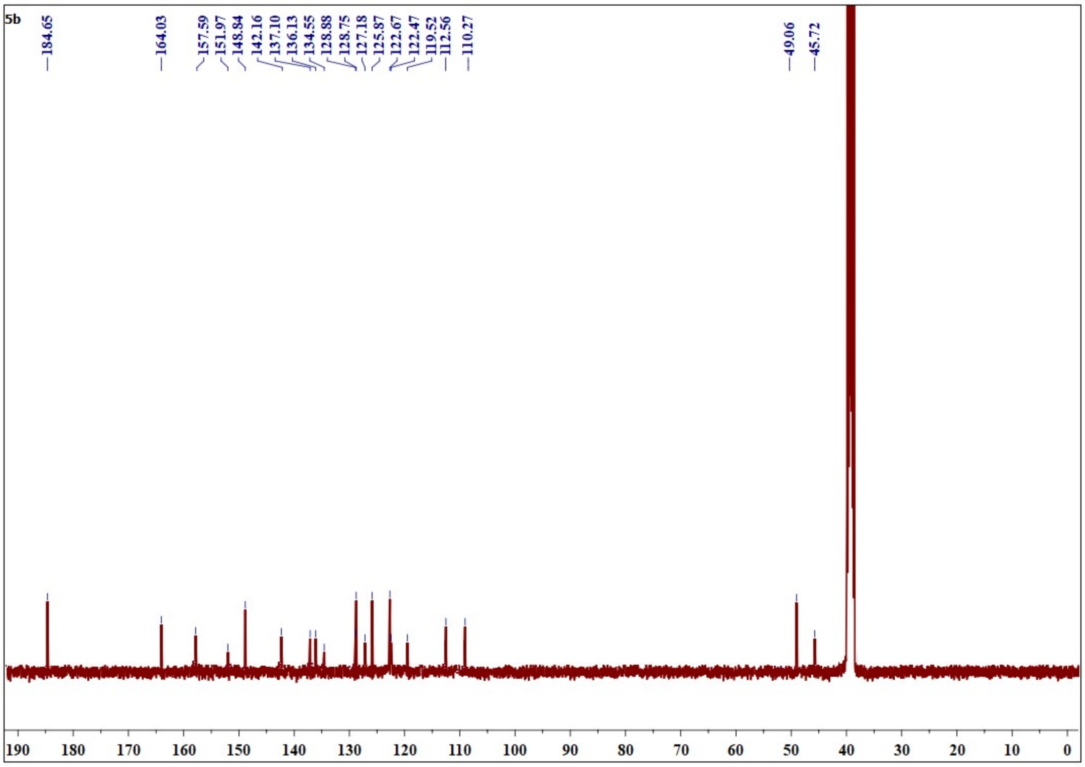

Figure S4. ^{13}C NMR spectrum of compound **5b** (100 MHz, DMSO-d_6).

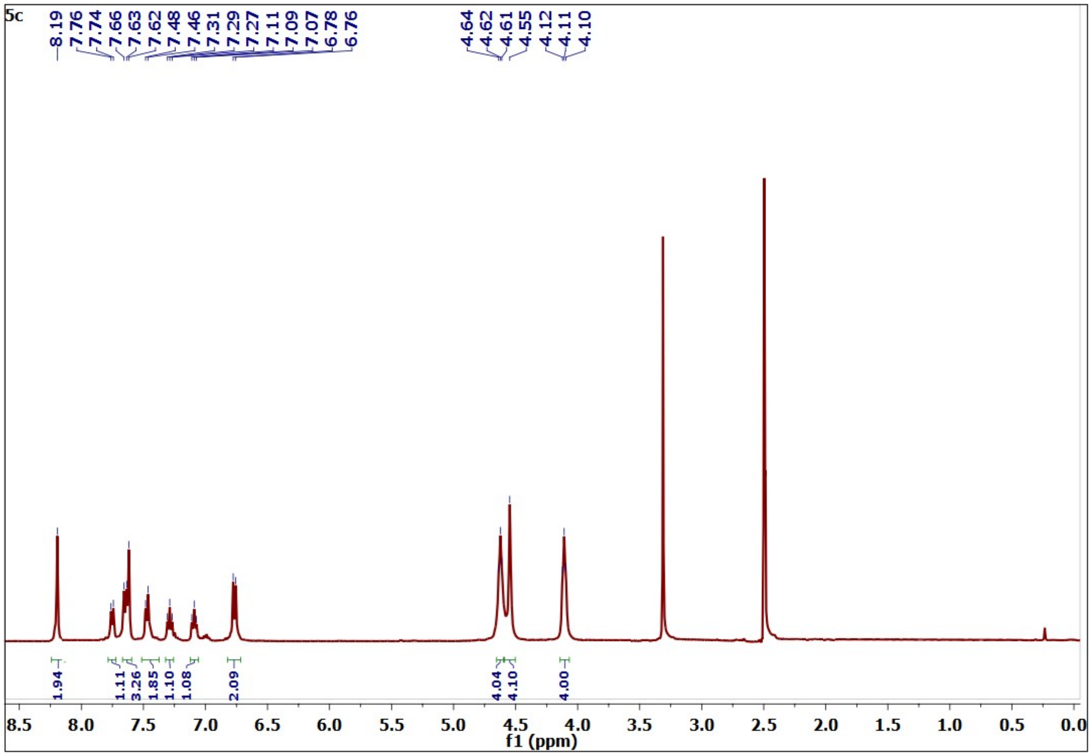

Figure S5. ^{1}H NMR spectrum of compound **5c** (400 MHz, DMSO-d_6).

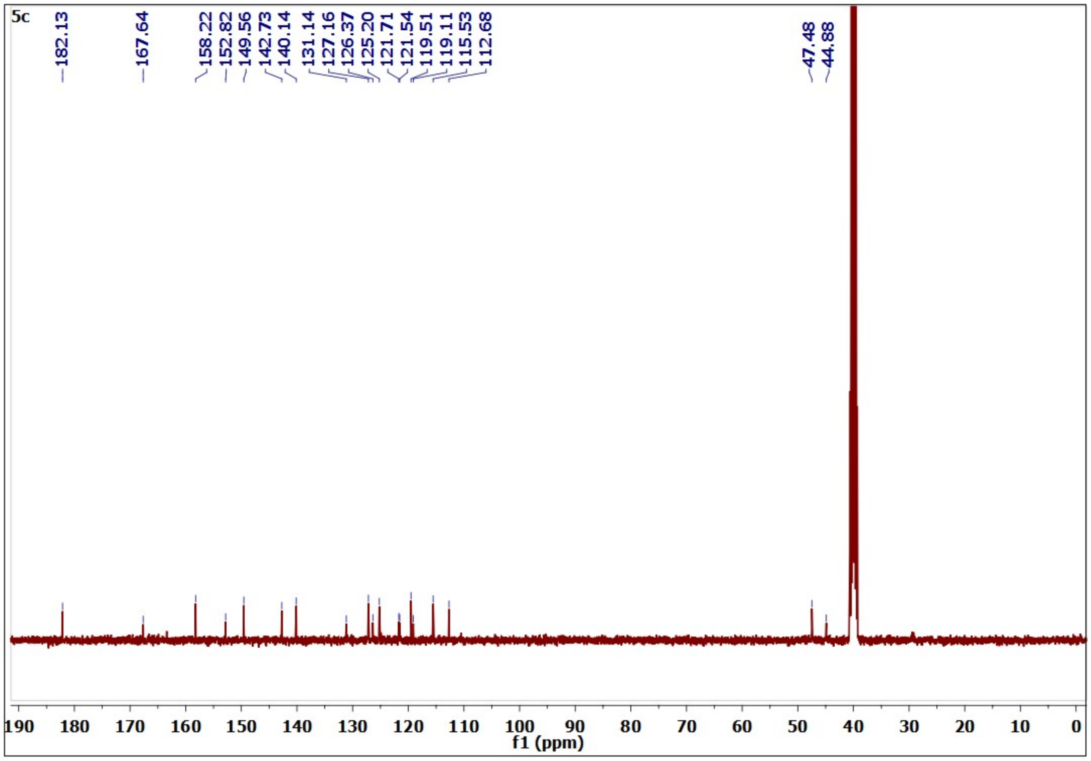

Figure S6. ^{13}C NMR spectrum of compound **5c** (100 MHz, DMSO-d_6)

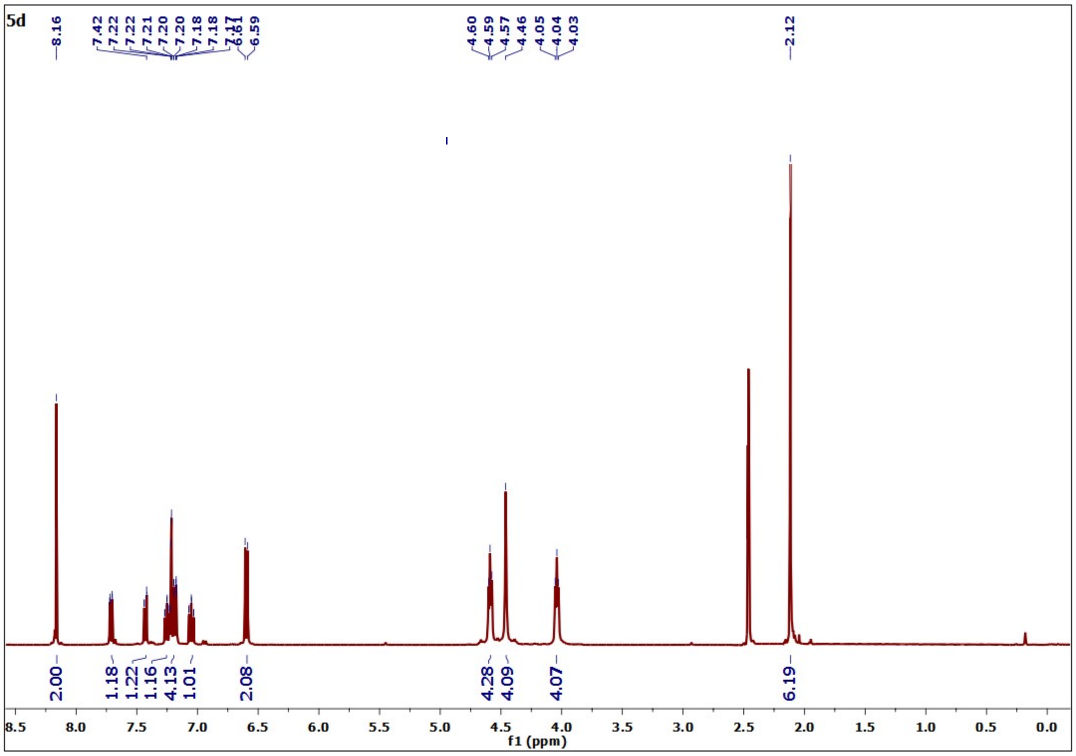

Figure S7. ^{1}H NMR spectrum of compound **5d** (400 MHz, DMSO-d_6).

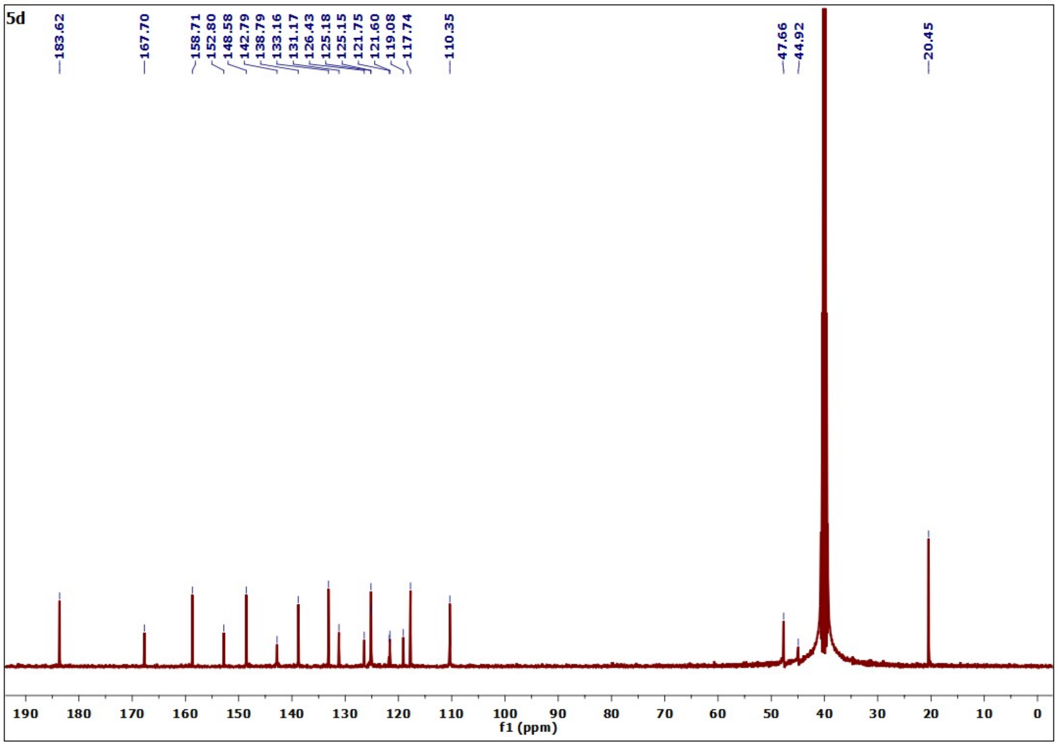

Figure S8. ^{13}C NMR spectrum of compound **5d** (100 MHz, DMSO-d_6)

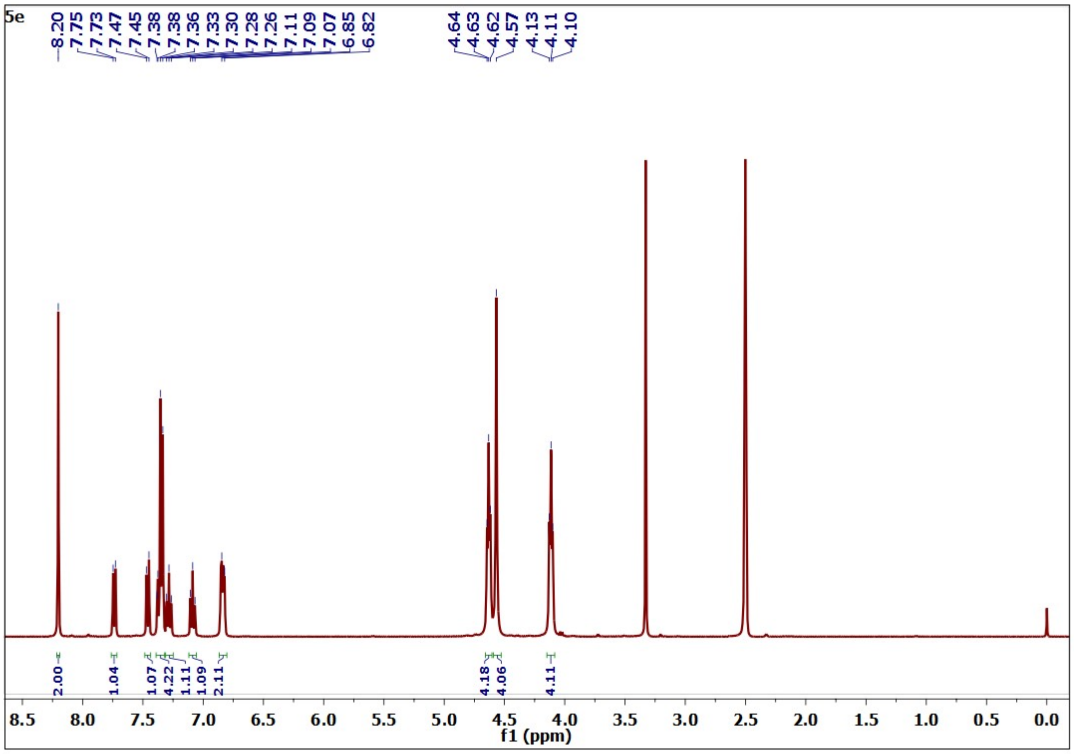

Figure S9. ^{1}H NMR spectrum of compound **5e** (400 MHz, DMSO-d_6).

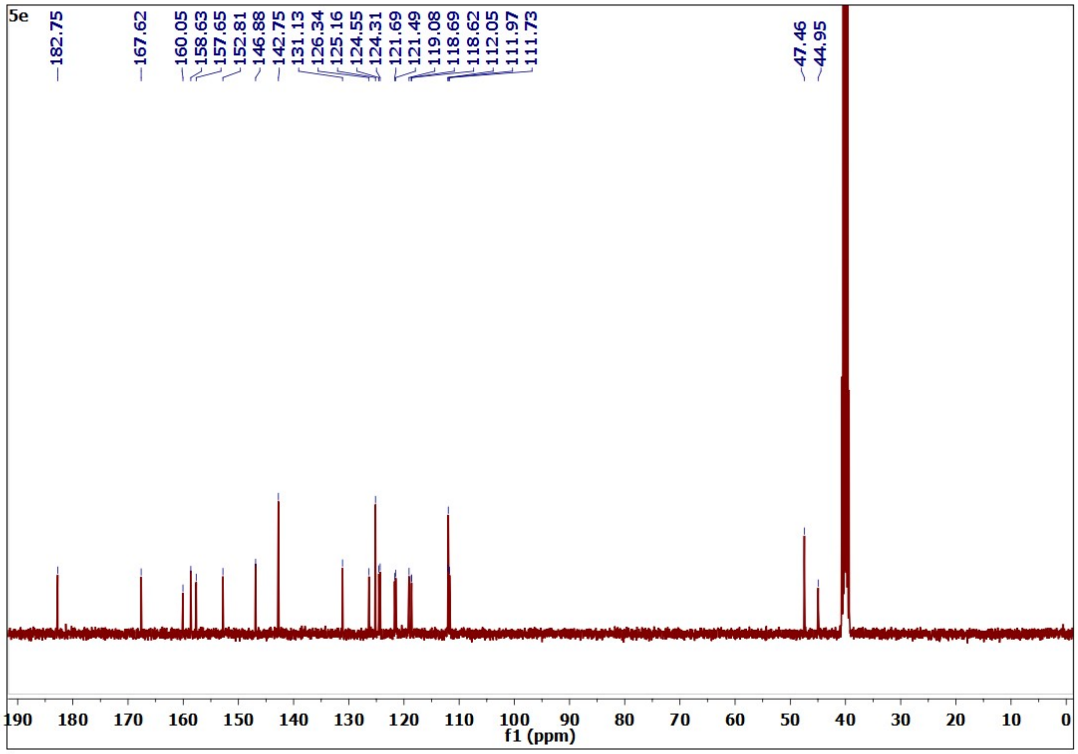

Figure S10. ^{13}C NMR spectrum of compound **5e** (100 MHz, DMSO-d_6)

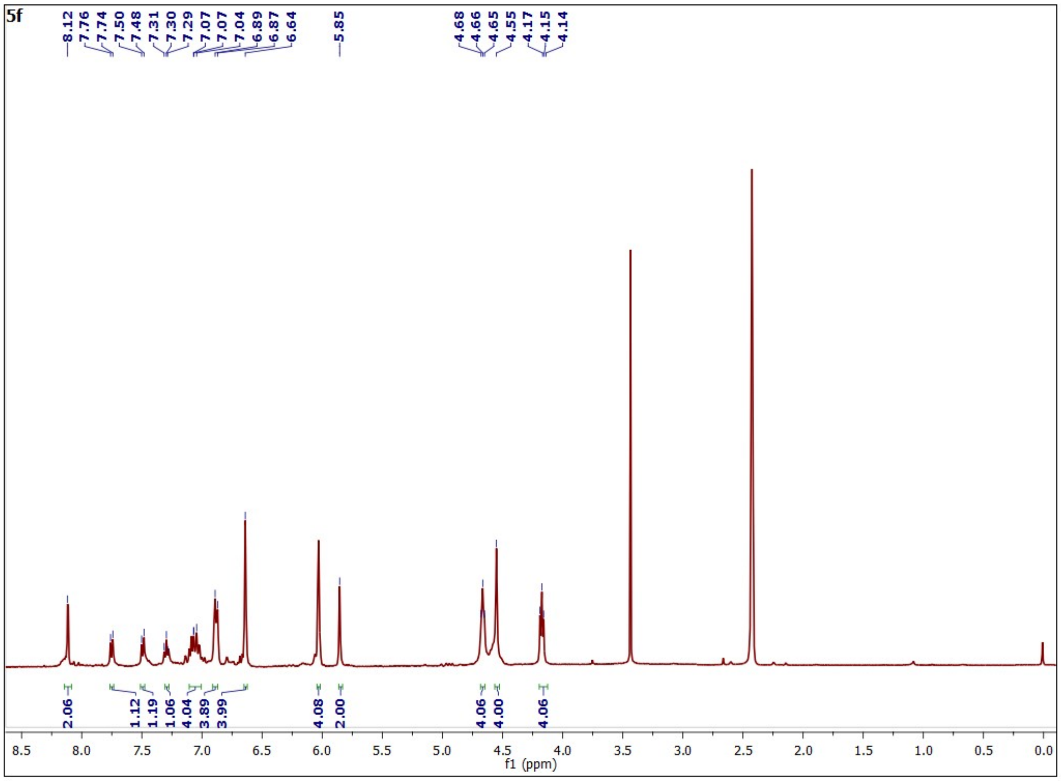

Figure S11. ^{1}H NMR spectrum of compound **5f** (400 MHz, DMSO-d_6).

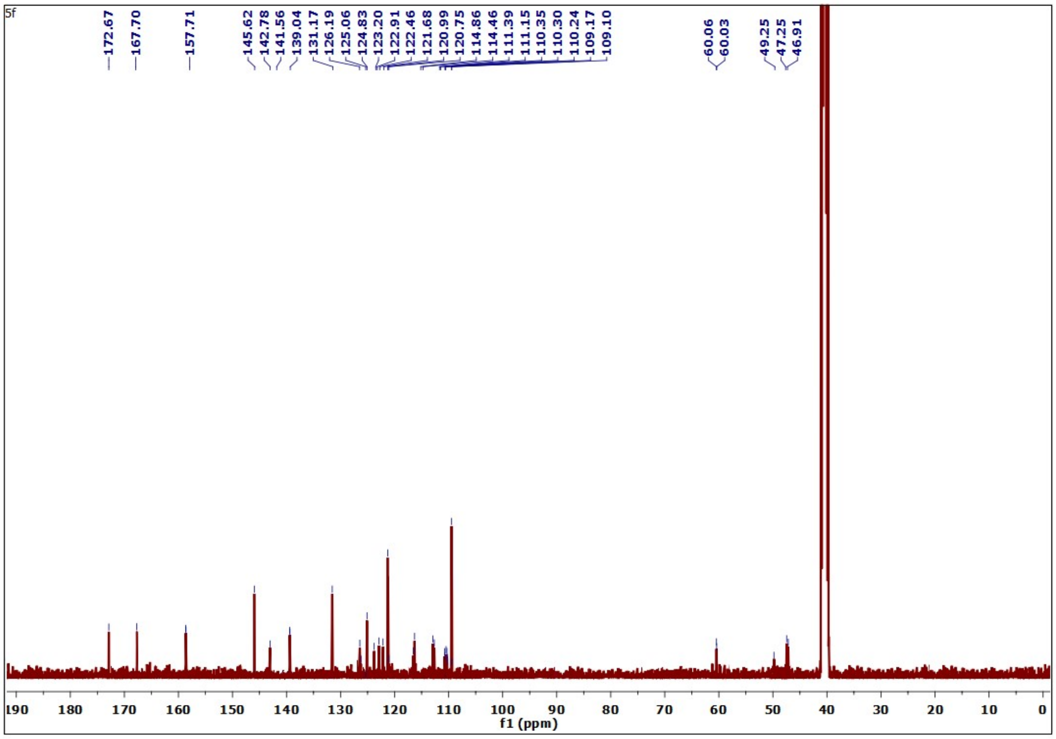

Figure S12. ^{13}C NMR spectrum of compound **5f** (100 MHz, DMSO-d_6)

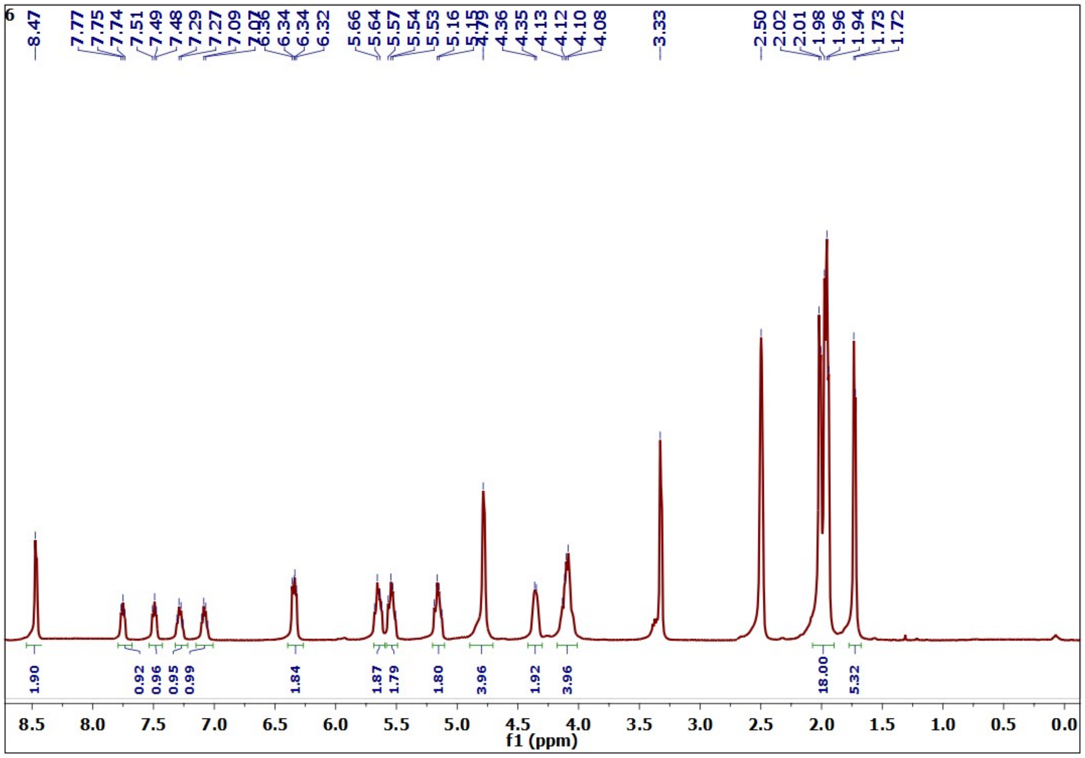

Figure S13. ^{1}H NMR spectrum of compound **6** (400 MHz, DMSO-d$_6$)

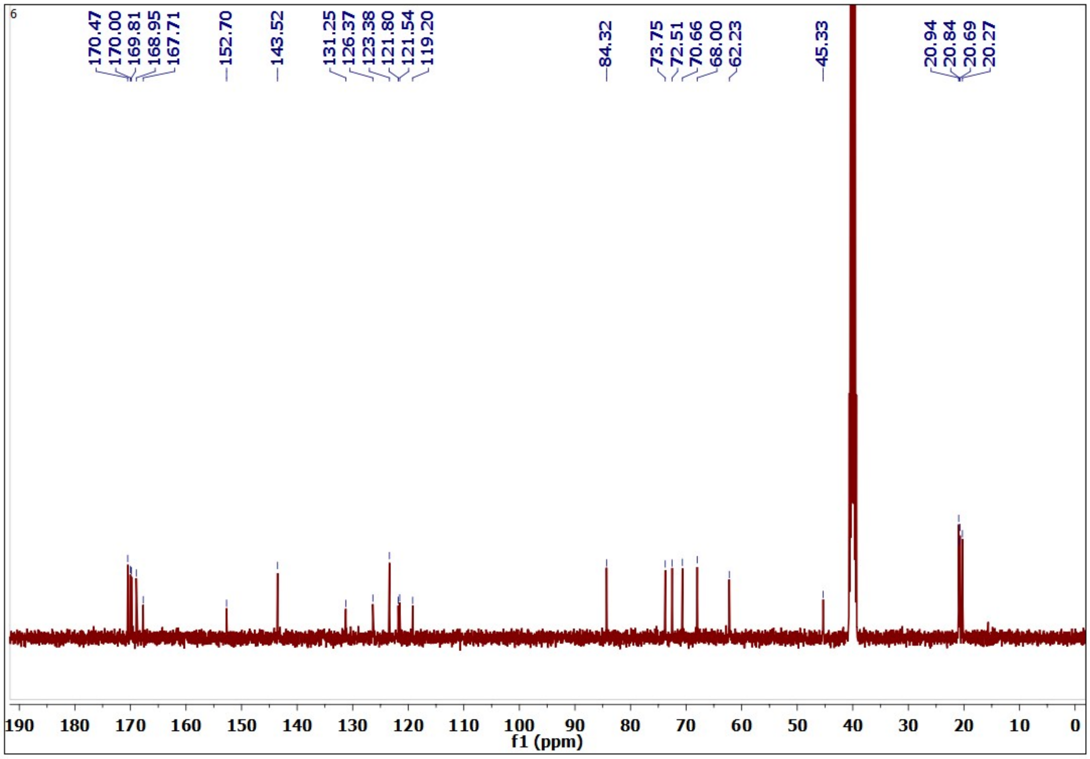

Figure S14. ^{13}C NMR spectrum of compound **6** (100 MHz, DMSO-d$_6$).

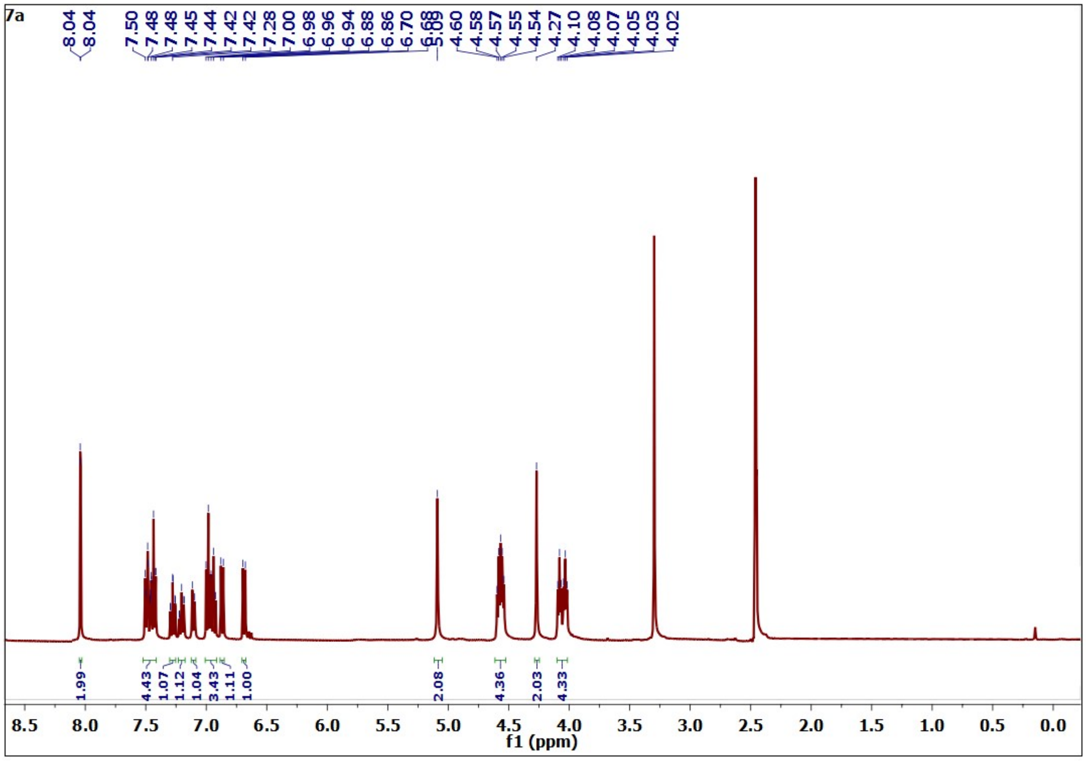

Figure S15. ^{1}H NMR spectrum of compound **7a** (400 MHz, DMSO-d_6).

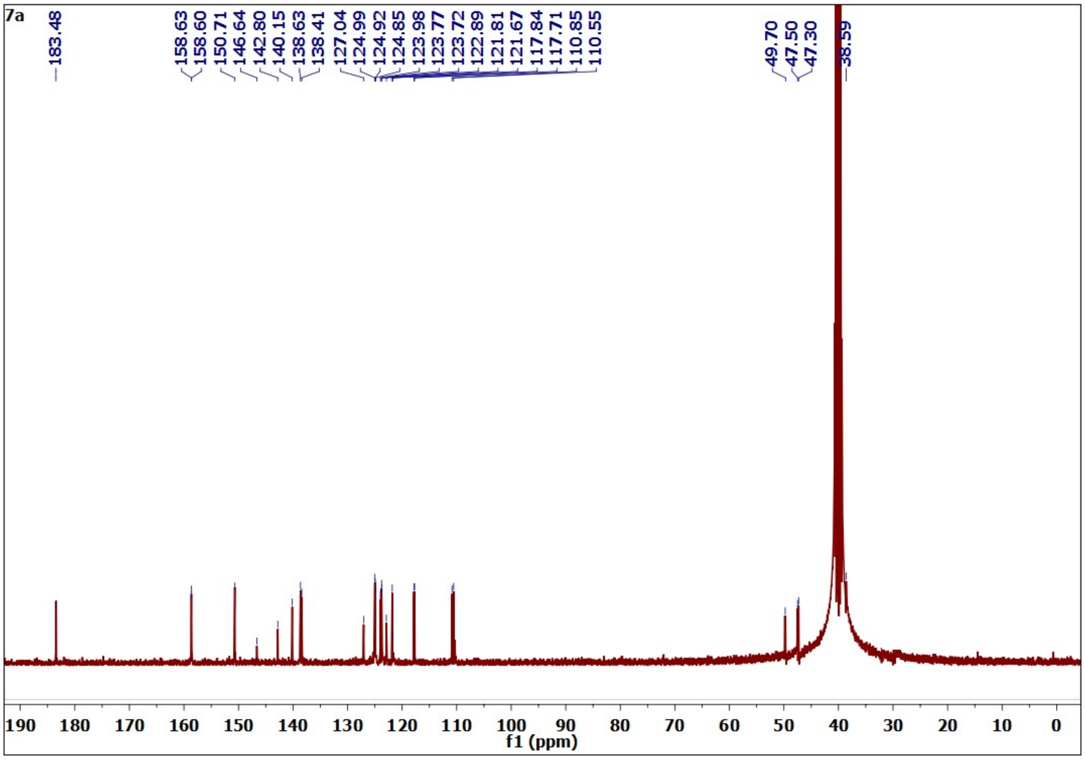

Figure S16. ^{13}C NMR spectrum of compound **7a** (100 MHz, DMSO-d_6)

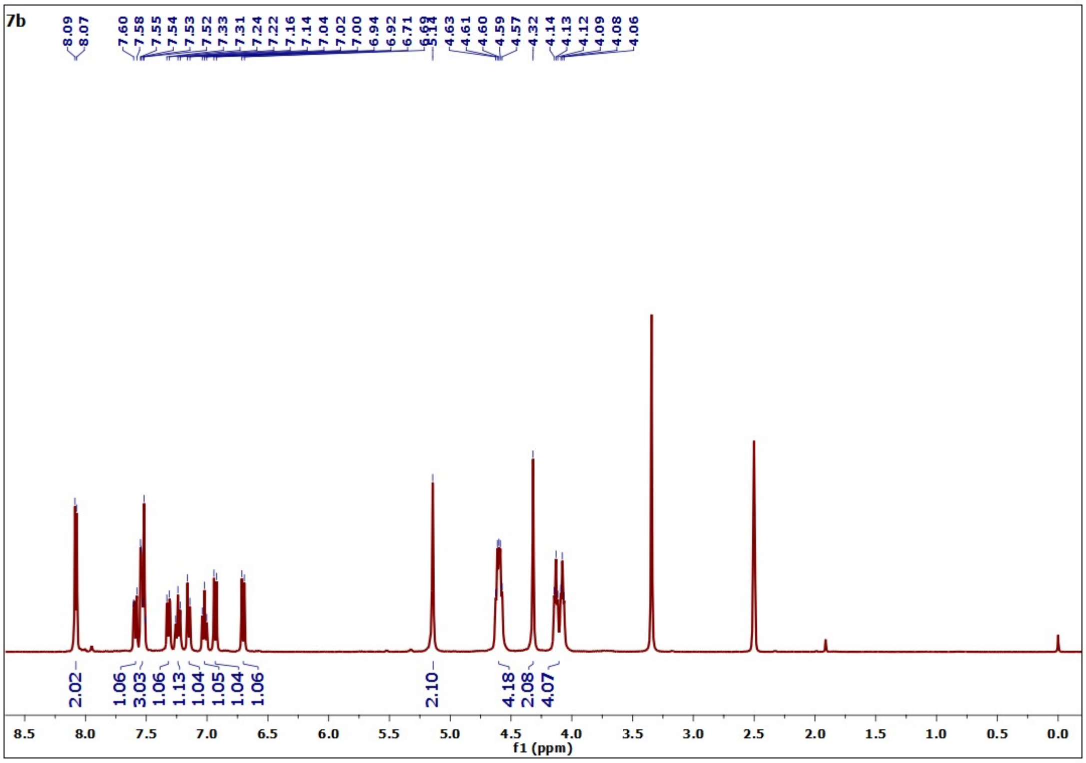

Figure S17. ^{1}H NMR spectrum of compound **7b** (400 MHz, DMSO-d_6).

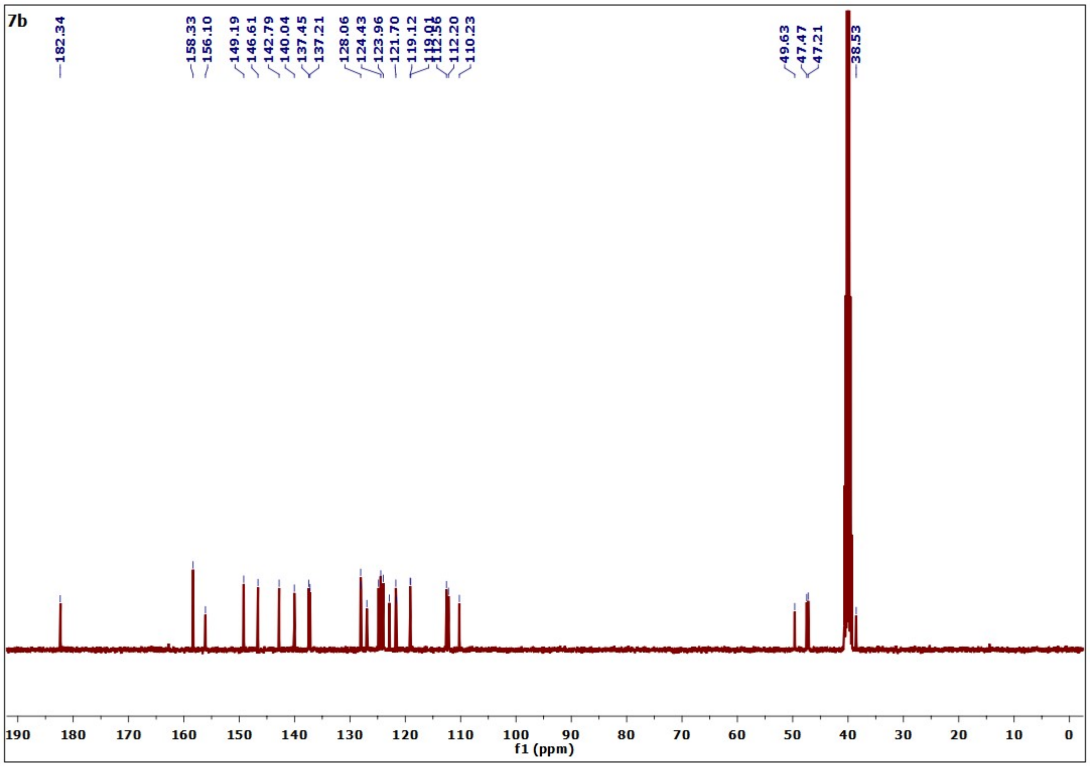

Figure S18. ^{13}C NMR spectrum of compound **7b** (100 MHz, DMSO-d_6).

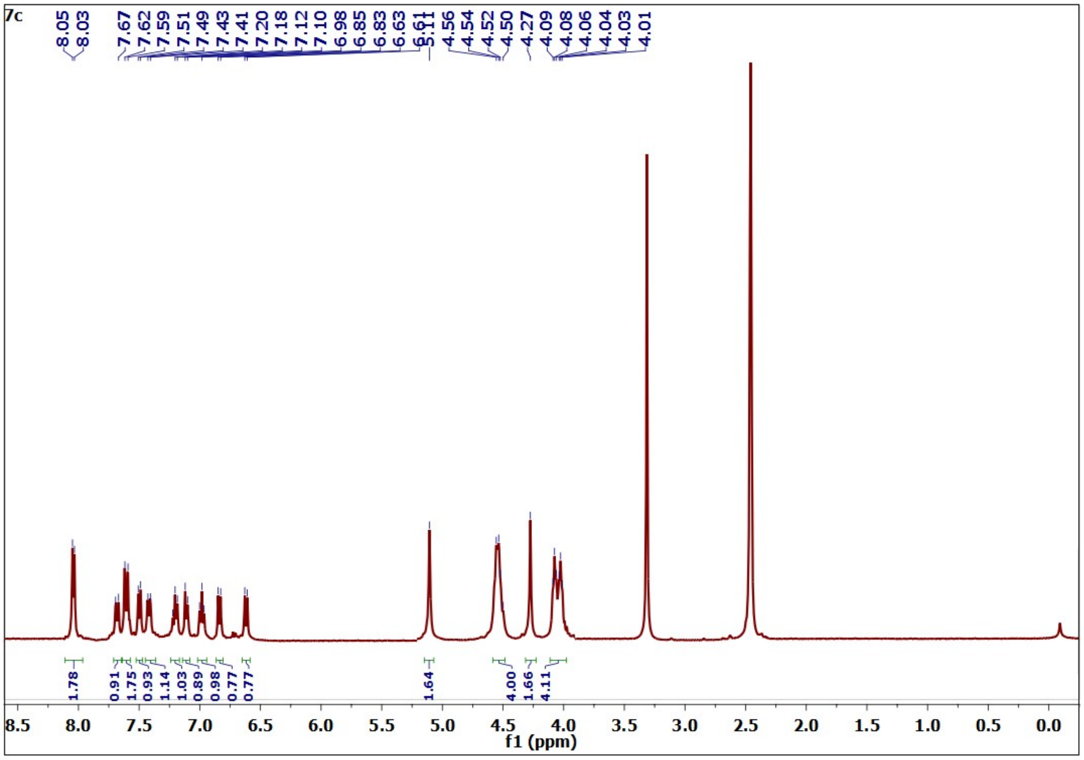

Figure S19. ^{1}H NMR spectrum of compound **7c** (400 MHz, DMSO-d_6).

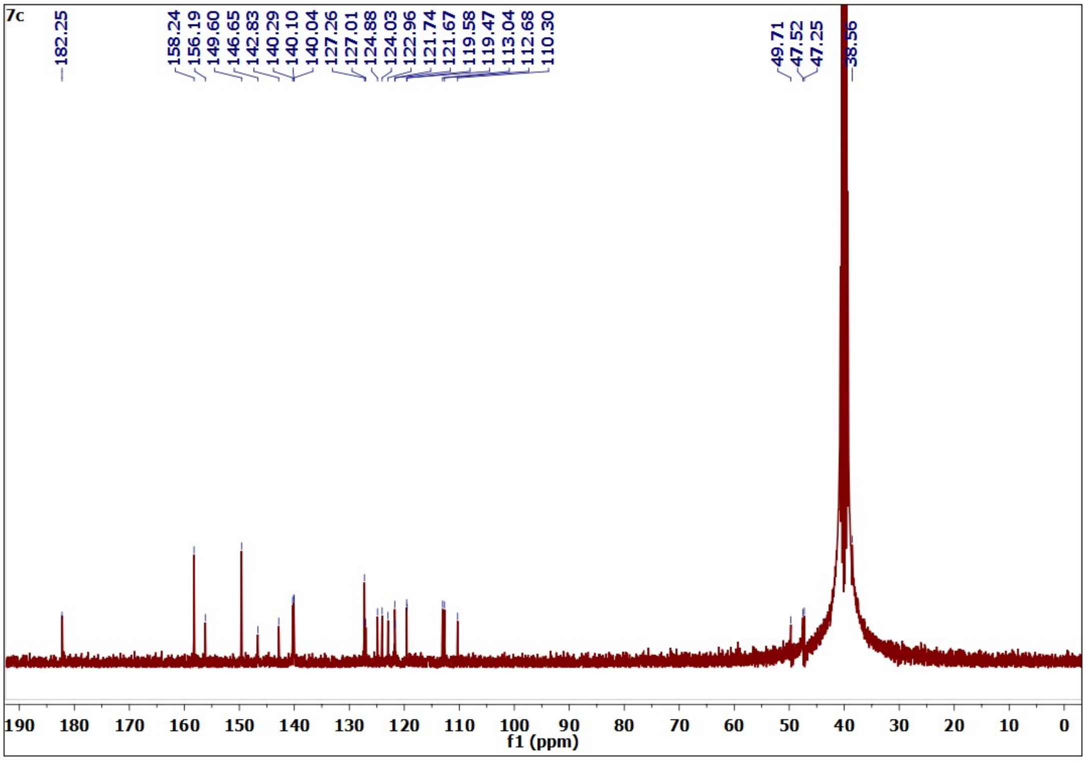

Figure S20. ^{13}C NMR spectrum of compound **7c** (100 MHz, DMSO-d_6)

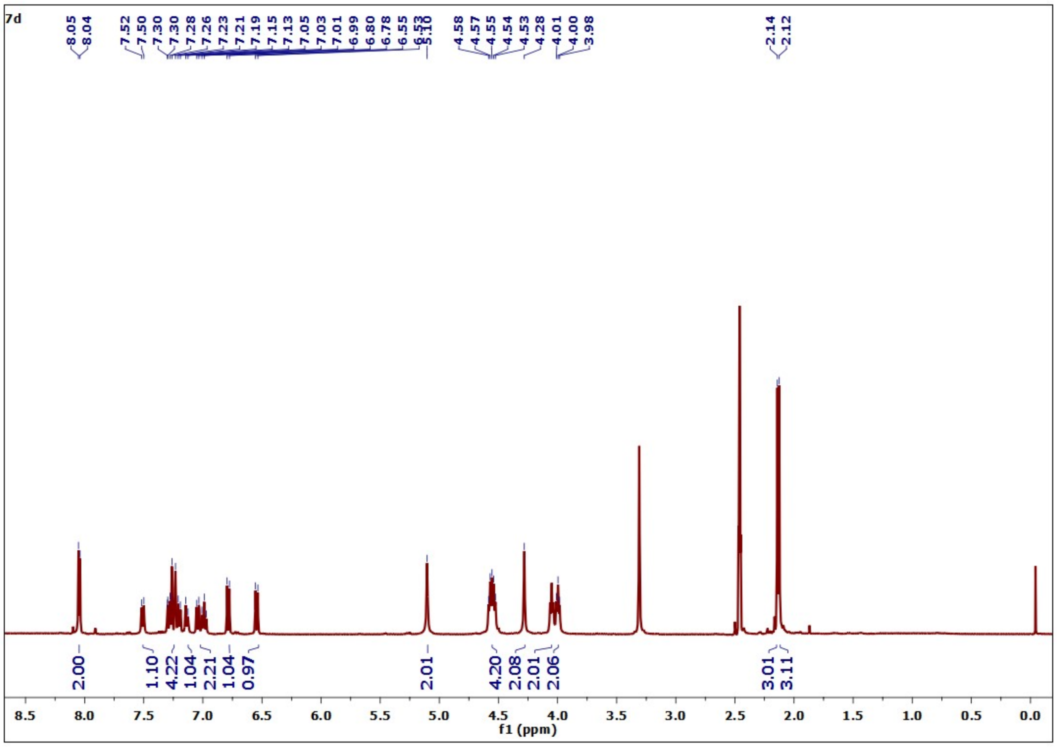

Figure S21. ^{1}H NMR spectrum of compound **7d** (400 MHz, DMSO-d_6).

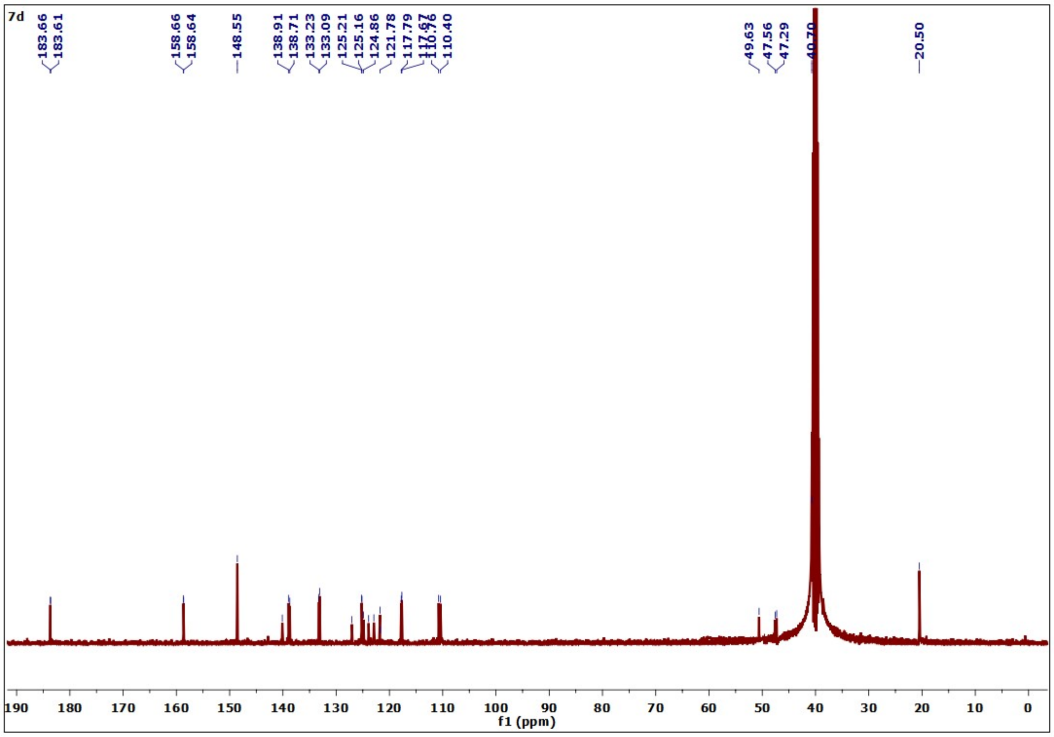

Figure S22. ^{13}C NMR spectrum of compound **7d** (100 MHz, DMSO-d_6).

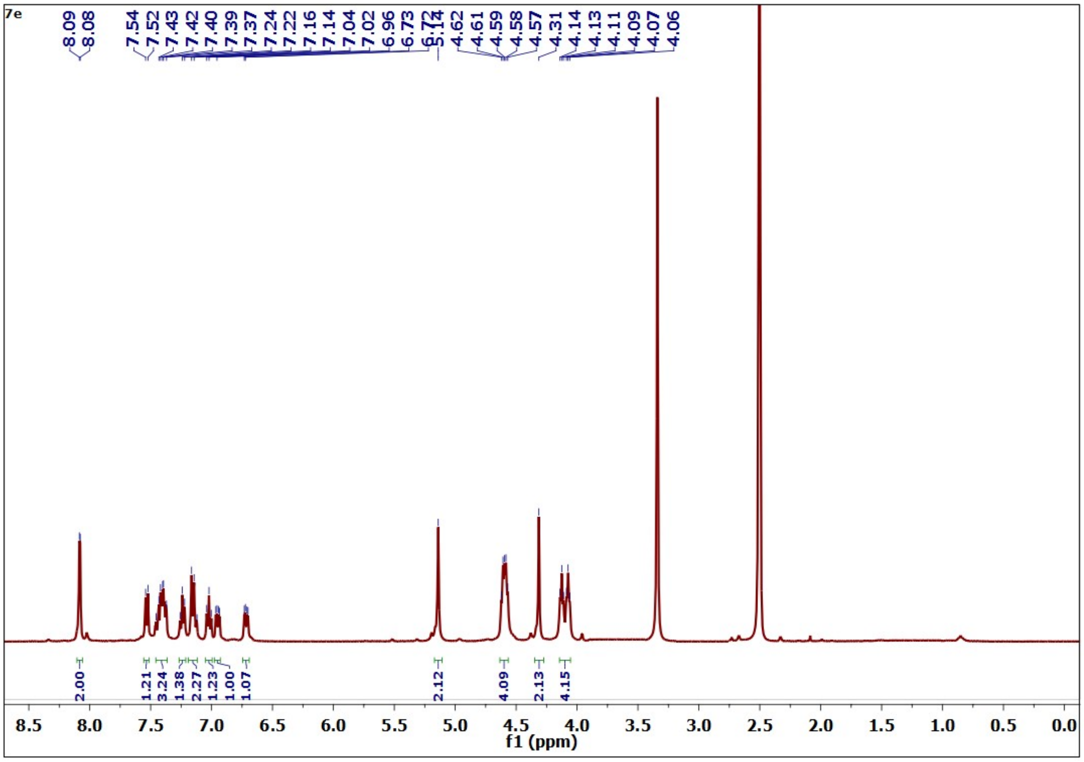

Figure S23. ^{1}H NMR spectrum of compound **7e** (400 MHz, DMSO-d_6).

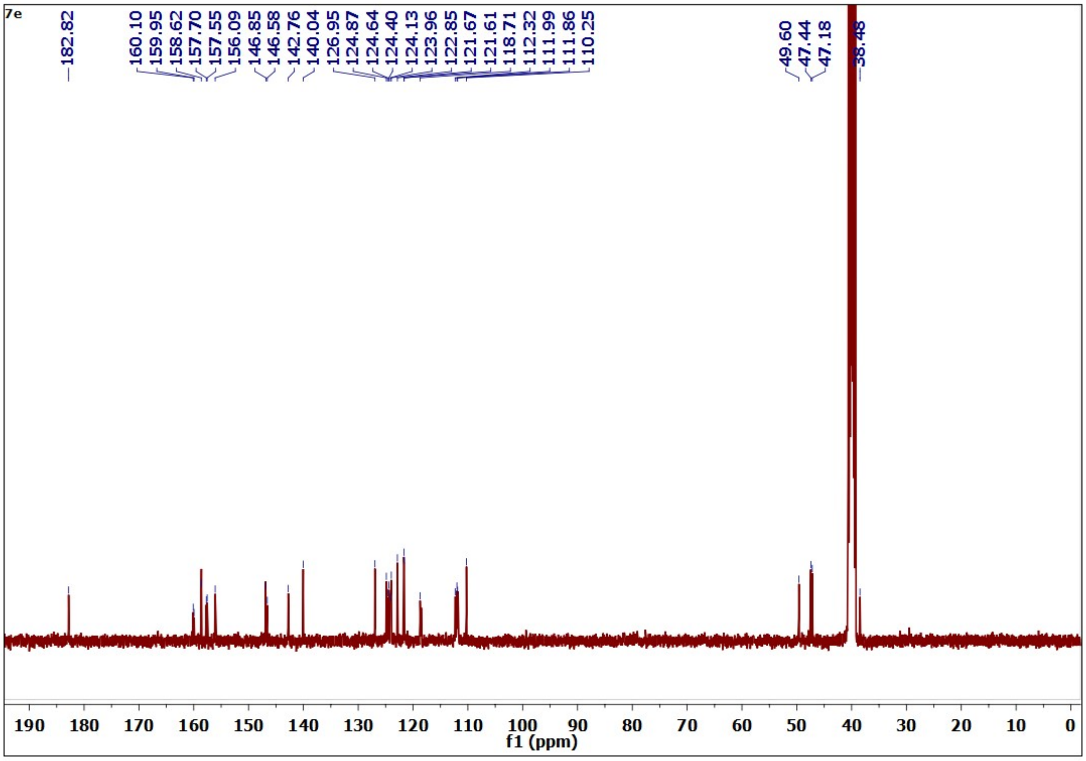

Figure S24. ^{13}C NMR spectrum of compound **7e** (100 MHz, DMSO-d_6).

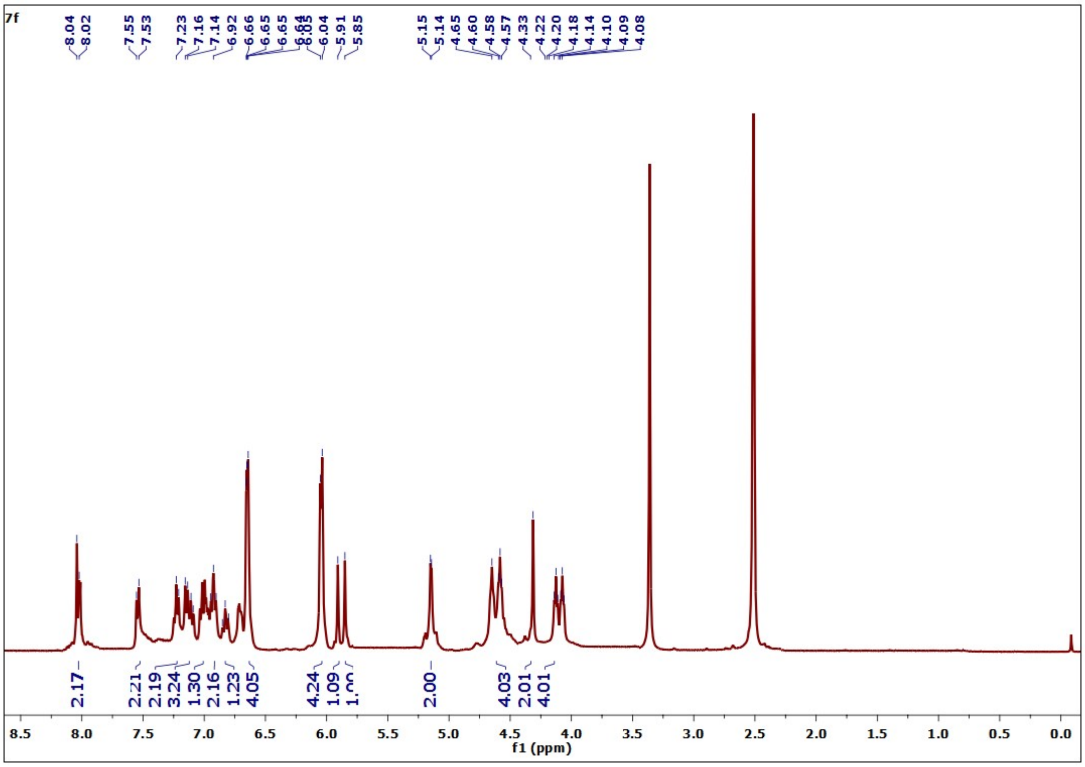

Figure S25. ^{1}H NMR spectrum of compound **7f** (400 MHz, DMSO-d_6)

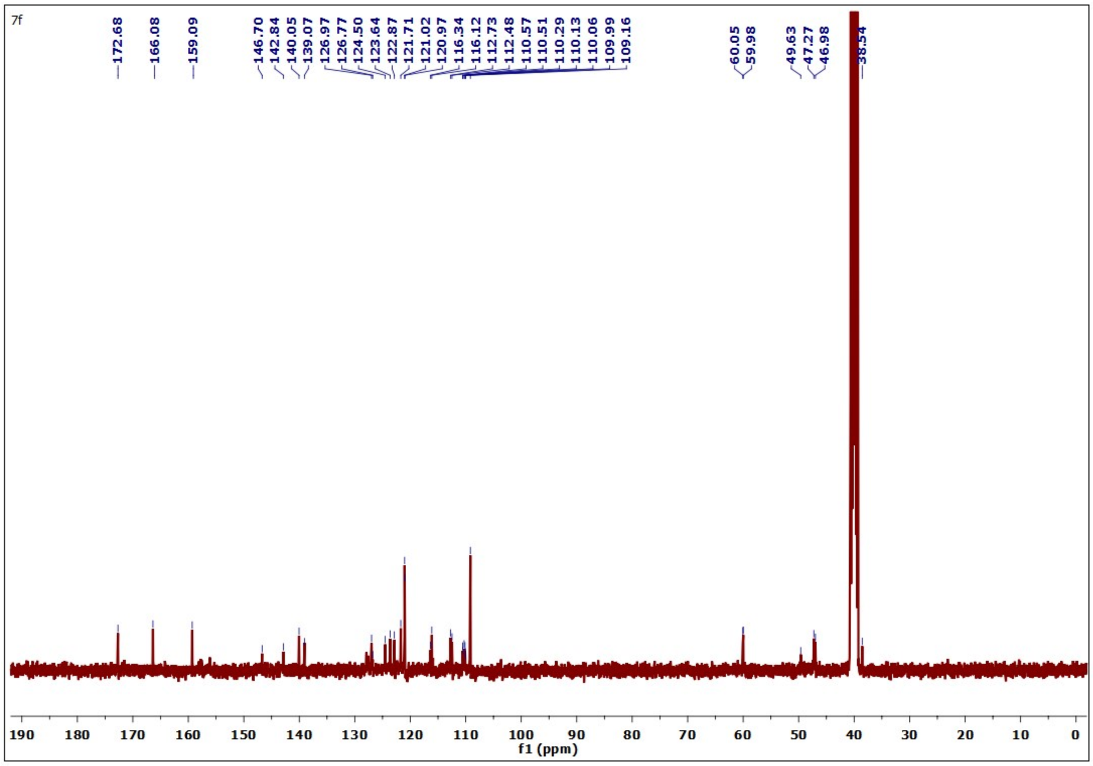

Figure S26. ^{13}C NMR spectrum of compound **7f** (100 MHz, DMSO-d_6).

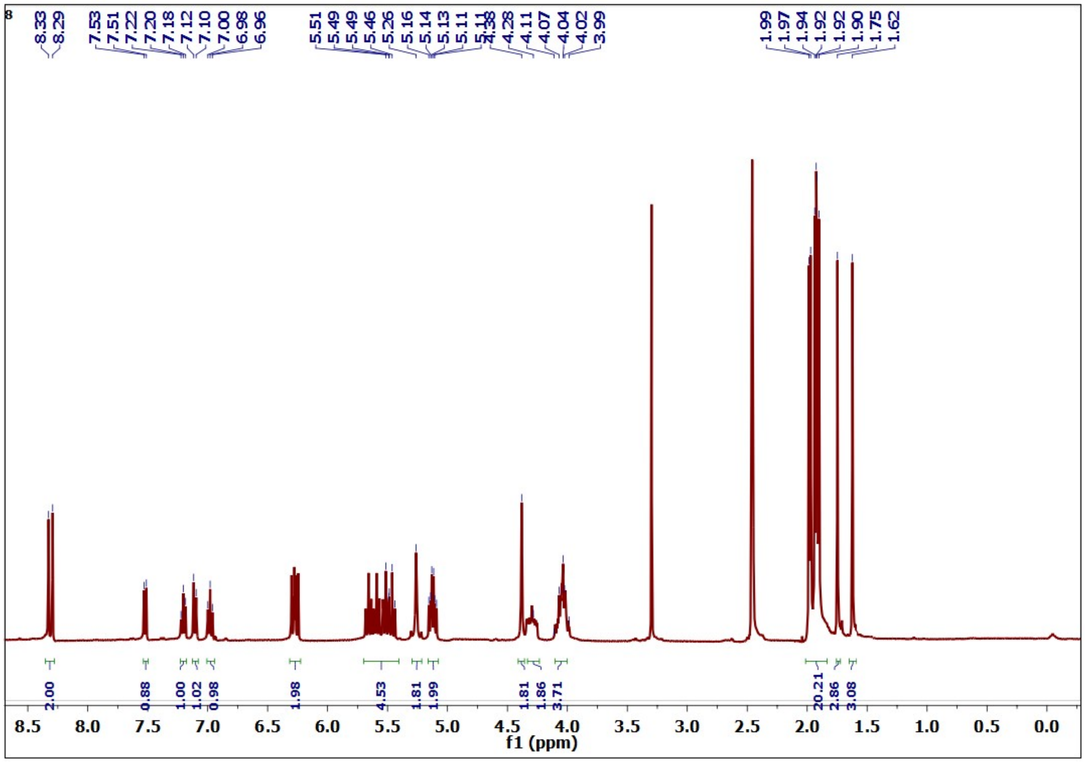

Figure S27. ^{1}H NMR spectrum of compound **8** (400 MHz, DMSO-d_6).

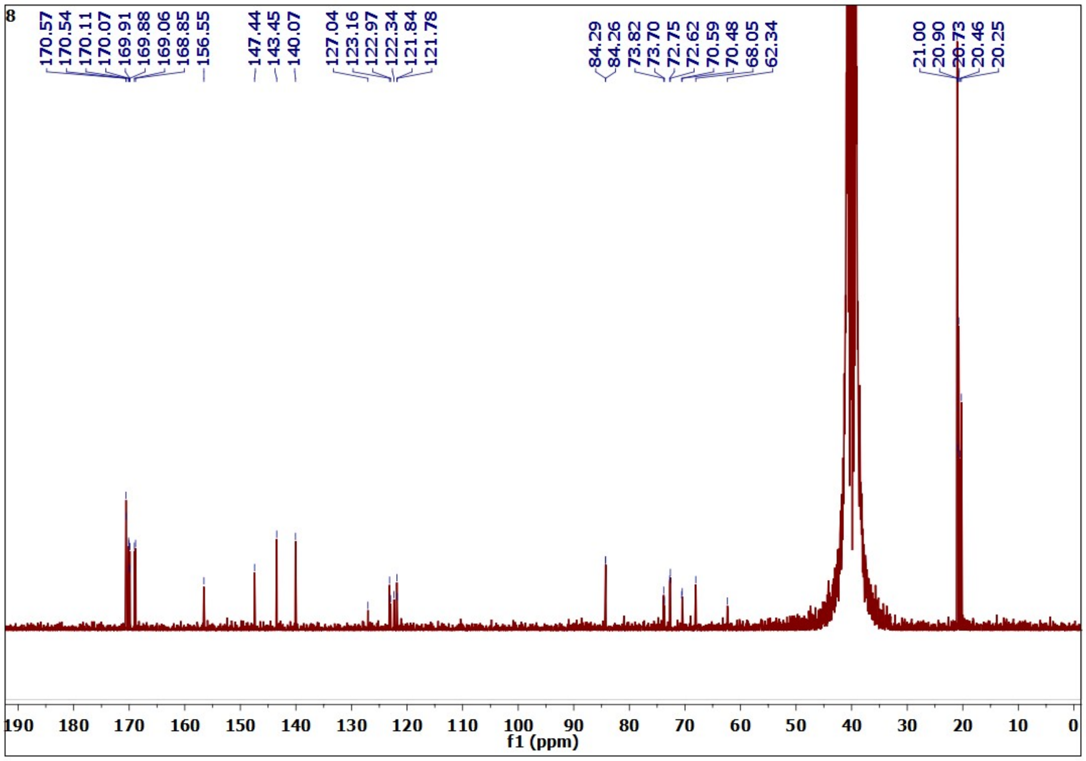

Figure S28. ^{13}C NMR spectrum of compound **8** (100 MHz, DMSO-d_6).
